# Magnetic MOF microreactors for recyclable size-selective biocatalysis†
†Electronic supplementary information (ESI) available: Experimental procedures, calibration curves and additional figures relating to capsule characterisation and biocatalysis. See DOI: 10.1039/c4sc03367a



**DOI:** 10.1039/c4sc03367a

**Published:** 2014-12-17

**Authors:** Jia Huo, Jordi Aguilera-Sigalat, Samir El-Hankari, Darren Bradshaw

**Affiliations:** a School of Chemistry , University of Southampton , Highfield Campus , Southampton SO17 1BJ , UK . Email: D.Bradshaw@soton.ac.uk ; Tel: +44 (0)23 8059 9076

## Abstract

In this contribution we report a synthetic strategy for the encapsulation of functional biomolecules within MOF-based microcapsules. We employ an agarose hydrogel droplet Pickering-stabilised by UiO-66 and magnetite nanoparticles as a template around which to deposit a hierarchically structured ZIF-8 shell. The resulting microcapsules are robust, highly microporous and readily attracted to a magnet, where the hydrogel core provides a facile means to encapsulate enzymes for recyclable size-selective biocatalysis.

## Abstract 2

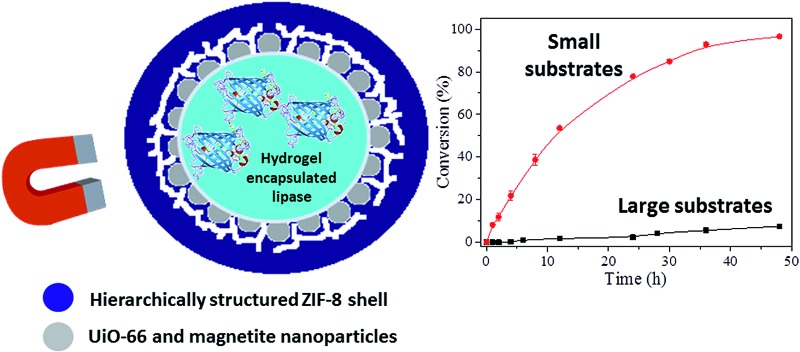
Highly porous magnetic MOF microreactors can be prepared around a Pickering-stabilized hydrogel core, providing a facile means for the encapsulation of enzymes for size-selective biocatalysis.

## Introduction

Biocatalysis with natural enzymes is extremely attractive for sustainable chemistry due to high selectivity and reactivity under mild reaction conditions, minimizing waste and energy input.^1^ However, these highly functional biological macromolecules do not always operate optimally in the organic solvents typically employed in synthetic chemistry and their recovery can be problematic.^2^ Immobilization or encapsulation of enzymes for biocatalysis is a proven strategy to overcome these limitations, and although a diverse range of materials have been employed to enhance enzyme activity and stability,^3^ the need to develop new methods and hybrid capsules with hierarchically structured shells, ordered porosity and controllable compositions is important to drive innovation in this and wider areas of biotechnology.

Metal–organic frameworks (MOFs) are crystalline microporous networks formed by the assembly of metal ions and organic linkers, which have high surface area and readily tuneable structures, pore sizes and compositions.^4^ While this ensures their widespread use in applications traditionally associated with porous materials (*e.g.* molecular storage, separation, delivery and catalysis),^5^ it also qualifies them as excellent potential supports for enzymatic biocatalysis. There remain few reports of immobilization of active enzymes onto MOF supports, and current strategies involve the adsorption of biomolecules into mesoporous^6^ and ultra-large pore frameworks,^7^ covalent surface modification of MOF crystals with proteins^8^ or host–guest interactions between tag-group(s) on the enzyme and MOF pores to anchor the large functional molecules to the surface.^9^ These post-synthetic methods are not without their drawbacks, and typically lead to significant reductions in porosity as the large protein molecules occupy pores or block (surface) apertures to the available framework microporosity; although not necessarily a barrier to function, this could be an issue for the selectivity and/or molecular diffusion of composite biocatalytic materials. Further, adsorption directly into MOF pores necessarily restricts the size of the proteins that can enter the pores^7^ which may need to undergo conformational changes^10^ that could compromise biomolecule activity. An alternative strategy to overcome these challenges is to configure MOFs into hollow microcapsular structures, where encapsulation of biomolecules within the capsule interior will not significantly impact upon the inherent porosity of the MOF shell and permit larger proteins to be used.

**Fig. 1:**
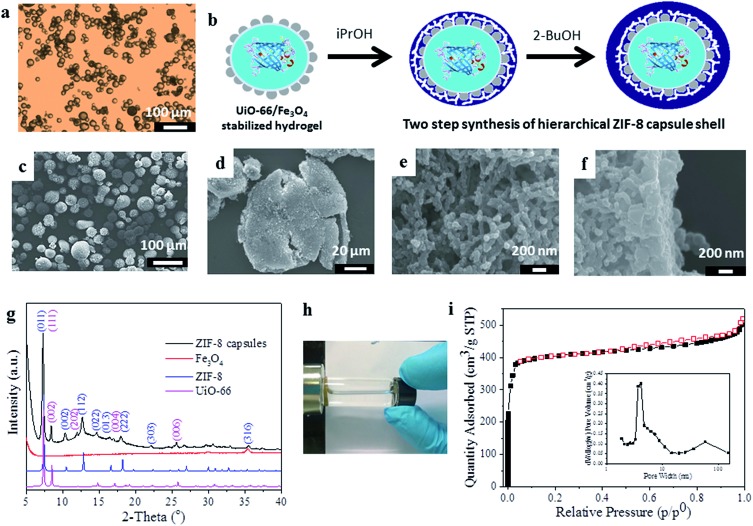
Synthesis and characterization of the magnetic MOF microcapsules. (a) Optical microscopy image of agarose hydrogel droplets Pickering-stabilized by UiO-66/Fe_3_O_4_ nanoparticles; (b) idealized representation of the two-step synthesis protocol to form the ZIF-8 shell, (c) SEM image of the intact capsules and (d) the crushed sample, showing their hollow nature and hierarchically structured shell comprising of (e) a network-like interior and (f) a dense outer-layer of ZIF-8; (g) PXRD data of the microcapsules compared to the single components, revealing the presence of both MOFs; (h) demonstration of the magnetic recovery of the capsules; (i) nitrogen adsorption–desorption isotherm of the capsules at 77 K (black squares, adsorption; red squares, desorption). The inset shows the BJH pore size distribution calculated from the adsorption branch of the isotherm.

MOF capsules have previously been prepared around sacrificial polystyrene cores,^11^ by interfacial synthesis in microfluidic devices,^12^ one-pot assembly around emulsion droplets^13^ and by spray-drying.^14^ None of these techniques however is amenable to the encapsulation of biomolecules: for example, solid sacrificial cores cannot readily encapsulate the desired cargo and the heat required to induce crystallization of a MOF shell or the presence of surfactant amphiphiles for the templating of hollow structures, have the potential to destabilize proteins. By contrast, we recently reported that MOF nanoparticles (NPs) can stabilize oil-in-water Pickering-type emulsions to form coordination-based analogues of colloidosomes, which can act as polymerization microreactors to form robust MOF-polymer composite capsules suitable for encapsulation and pH-triggered release of dye molecules.^15^ While there are several reports of Pickering stabilized biocatalysis microreactors using diverse particles^16^ and polymersomes,^17^ the exceptional microporosity and tuneable properties of MOFs provide a unique opportunity to develop novel potentially multifunctional hybrid composites with controllable structures, compositions and pore sizes. In this contribution we report a strategy for the encapsulation of functional biomolecules within MOF-based microcapsular structures. By utilizing a Pickering-stabilised hydrogel droplet we can deposit a hierarchically structured MOF shell to form robust free-standing capsules with high microporosity that are easily recovered by a magnet owing to a small amount of incorporated magnetite^18^ (Fig. 1). The hydrogel core provides a facile means to encapsulate large biomolecules under mild conditions that are not accessible to other MOF capsule formation methods. The resulting biomolecule loaded capsules can be employed for recyclable size-selective biocatalysis due to the high integrity of the microporous capsule shells, which regulate molecular access to and from the capsule lumen. As a direct consequence of the capsule structure and composition, we also find that enzyme activity is modestly enhanced compared to the control samples.

## Results and discussion

### MOF capsule formation and characterisation

For the encapsulation of enzymes within MOF capsules, we employ an inverse phase Pickering emulsion containing 1.5 wt% agarose hydrogel droplets in a continuous paraffin oil phase which are stabilized by pre-hydrophobized NPs of UiO-66^19^ [Zr_6_O_4_(OH)_4_(BDC)_6_, where BDC = 1,4-benzenedicarboxylate] and Fe_3_O_4_ (Fig. 1a). Heptanoic acid functionalised UiO-66 (Fig. S1†) was selected for its ready dispersibility in paraffin oil^15^ and to provide a surface for further MOF growth, and Fe_3_O_4_ to facilitate magnetic separation during capsule preparation and subsequent application.^18^ The average diameter of the spherical hydrogel droplets stabilized by 1 wt% added UiO-66/Fe_3_O_4_ NPs (wt/wt ratio 9 : 1) is 27.4 μm; however, this is tuneable by modulating the quantity of added particles (Fig. S2†).

As shown in Fig. 1b–f, a shell of ZIF-8,^20^ [Zn(2-MeIm)_2_] where 2-MeIm = 2-methylimidazole, is deposited around the UiO-66/Fe_3_O_4_ Pickering-stabilized hydrogel core. For ZIF-8 shell formation, we utilize a two-step synthesis protocol (Fig. 1b) that effectively separates out the framework nucleation and growth steps under solvent control at low temperature (–20 °C). The low reaction temperature improves overall shell integrity, maintains gelation of the core during shell formation and prevents deactivation of any incorporated biomolecules. The first step is conducted in isopropanol (^i^PrOH), and even at –20 °C leads to the rapid precipitation of ZIF-8 (Fig. S3†) which immediately provides additional stabilization to the UiO-66/Fe_3_O_4_ Pickering emulsion as ZIF-8 particles quickly aggregate around the hydrogel cores. This is followed by a slower growth-dominated step in 2-butanol (2-BuOH) (Fig. S3†) to yield highly robust free-standing capsules. Scanning electron microscopy (SEM) images reveal intact spherical structures of average size 37.6 ± 8.6 μm, which are demonstrably hollow when crushed (Fig. 1c and d and S4–S6†). The shell is 4–5 μm thick (by SEM) and has a clear hierarchical structure, reminiscent of supported MOF membranes.^21^ The interior of the shell is composed of a continuous mesoscale network of aggregated ZIF-8 NPs <100 nm in size (Fig. 1e, S5 and S6†), consistent with the observed very rapid nucleation of the framework. Overlaying this network-like interior is a thin (∼400 nm) but very dense coating of much larger and highly-intergrown ZIF-8 crystals (Fig. 1f), resulting from much slower framework growth in 2-BuOH. This dense over-layer gives the capsules additional structural stability, and strongly suggests these could find utility in size-selective applications where molecular access is controlled by the highly regular micropores of the ZIF-8 sodalite topology as previously reported for catalysis by ZIF-8 encapsulated noble metal NPs.^22^

The capsules were further characterized by powder X-ray diffraction (PXRD), indicating the presence of Bragg reflections corresponding to the cubic unit cells of both the deposited ZIF-8 shell and the UiO-66 NPs employed for emulsion stabilization (Fig. 1g), thus confirming they have a dual-MOF composition; this was also demonstrated by FTIR (Fig. S7†). A broad Bragg reflex of relatively low intensity observed at 2*Θ* ∼ 36° is assigned to the minor Fe_3_O_4_ component, which gives the capsules their slight brown/grey coloration and permits their ready attraction to a permanent magnet (Fig. 1h). The capsules have an overall composition of 1 : 32 : 63 for Fe_3_O_4_ : UiO-66 : ZIF-8 by weight as determined by ICP. Both MOFs are incorporated into the composite, and SEM images clearly reveal the presence of aggregated UiO-66 NPs clustered around the interior of the capsule shell (Fig. 1b and S6†).

Thermogravimetric analysis (TGA) indicates the capsules largely decompose in a single step in the temperature range 320–500 °C, and a small step is also observed between 500–650 °C (Fig. S8†). Nitrogen adsorption isotherms (Fig. 1i) at 77 K demonstrate the capsules are highly microporous with an apparent BET surface area of 1452 m^2^ g^–1^ and a pore volume of 0.82 cm^3^ g^–1^. A slight hysteresis is observed upon desorption, and application of a Barrett–Joyner–Halenda (BJH) pore size distribution analysis (Fig. 1i, inset) reveals pores across the meso- and macroporous regimes consistent with the hierarchical structure of the capsule shell observed by SEM (Fig. 1d–f). The capsules are highly stable, retaining their spherical shape upon solvent removal or exchange (Fig. S9†), and the MOF shell does not degrade after soaking in organic solvents, water or phosphate-buffered saline (PBS) (Fig. S10†) consistent with its composition.

### Capsule loading and shell integrity

**Fig. 2:**
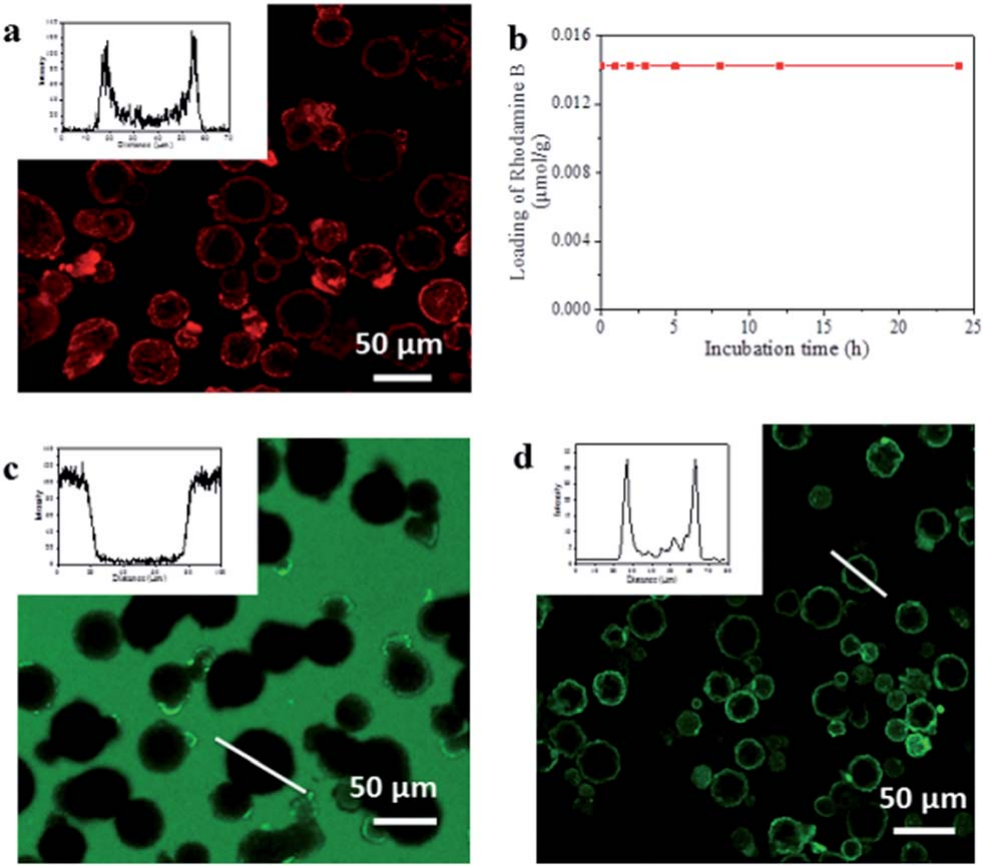
Encapsulation properties of the MOF composite microcapsules. (a) CLSM image of capsules loaded with RhB, and inset a typical fluorescence profile; (b) RhB retention *vs.* capsule incubation time in water at ambient temperature; (c) CLSM image and fluorescence intensity profile of the highlighted capsule (inset) after soaking capsules in methanolic solutions of FITC; (d) CLSM image of the capsules loaded with FITC-labelled CalB, and inset the fluorescence profile of the highlighted capsule.

To evaluate capsule shell integrity and hence their potential in size-selective applications, we used large dye molecules as fluorescent probes of molecular uptake and release (Fig. 2). Water soluble Rhodamine B (RhB) was loaded into the capsules by inclusion within the UiO-66/Fe_3_O_4_ stabilized hydrogel. Despite a rather low 0.58% encapsulation efficiency of RhB as a result of significant leaching from the hydrogel due to high solubility in the alcohols used for ZIF-8 shell formation, this remains sufficient to permit localization by confocal laser scanning microscopy (CLSM) and determine any leaching through the shell. The CLSM images in Fig. 2a reveal that the RhB is concentrated around and/or within the interior of the capsule shell as evidenced by the observed red halo and bimodal fluorescence intensity profile, consistent with a strong interaction between the shell and the dye.^23^ Control experiments with Fe_3_O_4_ only and UiO-66 only Pickering-stabilized hydrogels reveal a similar fluorescence profile for UiO-66, whereas a homogeneous distribution of RhB throughout the capsule lumen is observed when only Fe_3_O_4_ NPs are used (Fig. S11†). This confirms the role of the MOF particles in dye accumulation in the capsule shell, and since RhB is too large to enter the micropores of either ZIF-8 or UiO-66, this is most likely to be surface adsorption arising from interactions between the abundant organic functionalities of the hybrid framework particles and the aromatic dye.

Following acid dissolution of the capsules, the RhB loading level was determined as 0.014 μmol g^–1^ by UV/vis spectroscopy, which did not change after 24 h soaking in water (Fig. 2b) confirming the intact nature of the capsule shell. The microcapsules were also immersed in dye solutions to investigate diffusion into the interior from bulk solution. CLSM images show the interior and shell of the capsules remain totally dark when soaked in methanolic solutions of fluorescein isothiocyanate (FITC) (Fig. 2c) or aqueous RhB (Fig. S12†). By contrast capsules isolated after step one of the synthesis in ^i^PrOH only, show significant FITC uptake into the meso-/macroporous network-like shell structure (Fig. S13†) verifying the importance of the intact microporous ZIF-8 outer layer to control molecular diffusion into and out of the capsules.

The UiO-66/Fe_3_O_4_ stabilized hydrogel core provides a facile means for the encapsulation of diverse and functional biomolecules into the robust MOF microcapsules. We investigated this by adding green fluorescent protein (GFP) and the FITC tagged enzymes Candida Antarctica lipase B (CalB) and β-galactosidase (β-gal) to the Pickering-stabilized agarose hydrogels prior to shell formation. CLSM images of the loaded capsules (Fig. 2d and S14†) clearly demonstrate successful encapsulation of the fluorescent proteins and reveal their accumulation around and/or within the interior of the capsule shells in a similar manner to the dyes. This likely arises from the composition of the MOF shell, which appears to display an appropriate hydrophilic/phobic balance to enhance interactions with encapsulated biomolecules. To investigate whether the protein molecules were immobilized within interstices between the aggregated NPs which comprise the interior of the hierarchically structured shell, CalB loaded capsules were incubated in more polar MeOH at r.t. overnight. CLSM reveals the fluorescent enzyme becomes homogeneously distributed throughout the lumen (Fig. S15†), indicating permeability of the shell to small molecules and solvent dependent enzyme mobility within the capsules.

The proteins employed in this study range in molecular weight from 27 kDa for GFP to 465 kDa for the large homo-tetrameric enzyme, β-gal. While some reports of protein incorporation into MOFs do exist,^6,7^ even the current largest pore frameworks (*viz.* IRMOF-74-XI which has 10 nm pores) will be unable to accommodate such large molecules as β-gal which has dimensions of 3.0 × 13.5 × 17.5 nm; hence the configuration of MOFs into capsules or other appropriate superstructures^24^ under mild conditions as demonstrated here is clearly necessary to meet these demands.

### Biocatalysis

**Fig. 3:**
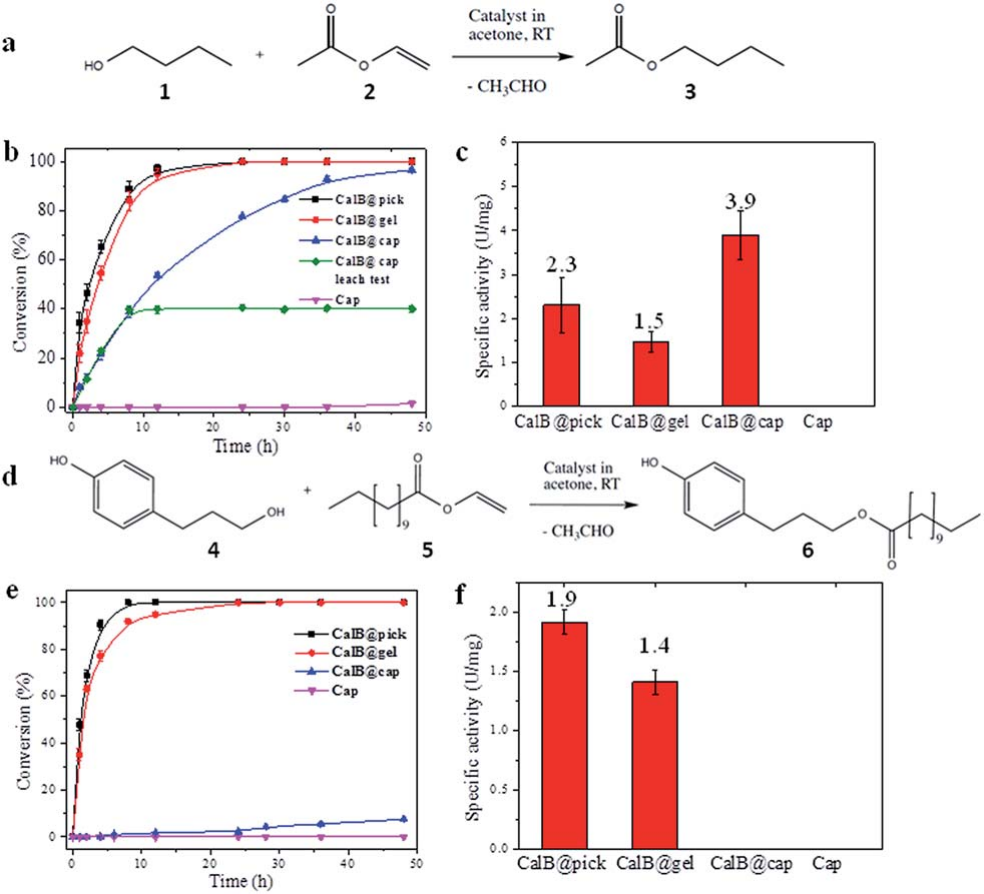
Biocatalysis using CalB loaded MOF microcapsules. (a) Reaction scheme for the transesterification between 1-butanol (**1**) and vinyl acetate (**2**) to yield butyl acetate (**3**); (b) observed time dependent conversion of **1** and **2** catalyzed by CalB under different conditions (CalB@pick, black; CalB@gel, red; CalB@cap, blue; cap, purple; the data in green shows conversion following removal of CalB@cap after 10 hrs); (c) normalized activities (U = μmol min^–1^) of the capsules and controls for the formation of **3**; (d) scheme of transesterification between 3-(4-hydroxyphenyl) propan-1-ol (**4**) and vinyl laurate (**5**) to yield 3-(4-hydroxyphenyl)propyl dodecanoate (**6**). (e) Observed time dependent conversion of **4** and **5** catalyzed by CalB under different conditions (CalB@pick, black; CalB@gel, red; CalB@cap, blue; cap, purple); (f) normalized activities of the capsules and controls for the formation of **6**.

CalB is a robust broad-substrate lipase (MW = 33 kDa) widely employed for the synthesis of organic compounds through esterification and transesterification reactions in organic solvents,^25^ making it an excellent model candidate for microencapsulation and biocatalytic studies (Fig. 3). CalB loaded microcapsules (CalB@cap) were prepared as described, and were found to maintain a very high degree of porosity (BET = 1438 m^2^ g^–1^) (Fig. S16†) unlike previous studies where enzymes have been physically adsorbed into MOF mesopores^6,26^ or covalently immobilized on their crystal surfaces,^8^ strongly indicating that encapsulation is a superior strategy to prepare highly porous MOF-enzyme composite materials. Following capsule degradation, a Bradford assay revealed a CalB loading of 7 mg g^–1^ (0.2 μmol g^–1^), comparable to the level observed for enzyme immobilization on some mesoporous silicas^27^ but lower than reported for direct adsorption into MOF mesopores over a two day period.^6^ The maximum loading level of CalB into the capsules has not been fully explored, but inclusion of a higher concentration into the Pickering-stabilized hydrogel and further optimisation of the encapsulation process is expected to lead to increased enzyme loading.

The biocatalytic performance of CalB@cap and the size selectivity of the microcapsule shell were assessed by carrying out transesterification reactions between a pair of small substrates and a pair of larger ones as shown in Fig. 3a and d, respectively, and Fig. S17.† The following experiments were also performed: CalB in agarose gel droplets Pickering-stabilized by UiO-66/Fe_3_O_4_ (CalB@Pick) and CalB in agarose only (CalB@gel) as positive controls and empty MOF microcapsules (cap) as a negative control (Fig. S18†). Biocatalysis reactions were carried out at r.t. in acetone and progress monitored by ^1^H NMR; products were further identified with ESI-MS. For reaction of **1** and **2** (Fig. 3a), the positive controls reach 100% conversion to **3** after 12 h whereas CalB@cap takes ∼48 h (Fig. 3b and S19–S23†). This indicates enzyme activity is maintained following ZIF-8 shell formation and suggests that **1–3** can readily diffuse through the framework micropores of the intact outer shell. Magnetic removal of the capsules from the reaction after 10 h reveals no further conversion to **3**, demonstrating CalB is not leached from the microcapsules which remain fully intact and impermeable to FITC (Fig. S24†) following catalysis. As anticipated, the empty microcapsules without CalB are inactive for transesterification under these conditions. The capsules have a CalB loading efficiency of 30% (Bradford assay) resulting from incomplete shell formation during synthesis, and while the reduced enzyme contents largely account for the shallower conversion-time profile observed for CalB@cap, a contribution due to diffusion through the capsule shell will certainly play an important role.

Following normalization to the total protein contents (Fig. S25†), the specific activity (U mg^–1^) of CalB@cap is 2.6 times higher than CalB@gel and 1.7 times greater than CalB@Pick (Fig. 3c). This is attributed to differences in interfacial area, as commonly observed in Pickering-derived microreactors.^17,28^ CalB@gel displays the lowest specific activity due to the biphasic nature of the reaction where the substrate solution sits above a hydrogel monolith (Fig. S18†). Activity is increased by a factor of 1.5 through Pickering stabilization of the hydrogel droplets with UiO-66/Fe_3_O_4_ NPs, which present a larger interface for reaction (Fig. S18†). The highest activity is consequently observed for CalB@cap resulting from the hierarchically structured microcapsule shell, as previously reported for polydopamine capsules with highly interconnected porous wall structures.^29^ There is also likely to be an enzyme stabilizing effect by the capsule shell through hydrophobic interactions, and the potential to accumulate substrate molecules around CalB *via* MOF micropores may further contribute to the observed enhancement of activity. The transesterification of **1** and **2** was also carried out in hexane, where the specific activity of CalB@cap was less than that observed in acetone revealing that solvent effects remain important to the encapsulated biocatalysts. Activity of CalB@cap in hexane was also enhanced by a factor of 5 relative to the free enzyme (Fig. S26†), further confirming the role of the MOF shell in enzyme stabilization.

**Fig. 4:**
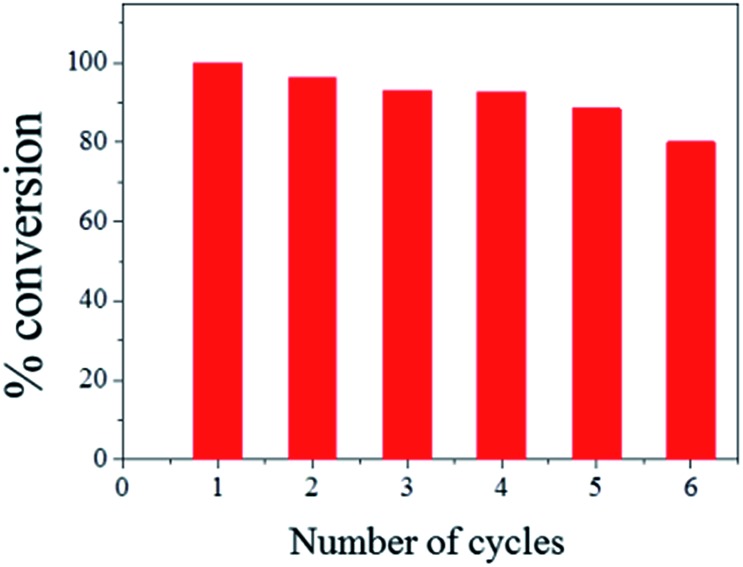
Catalytic recyclability of CalB@cap for the transesterification of **1** and **2** in acetone. The catalyst was removed following 8 h of reaction and conversion to **3** calculated by ^1^H NMR for each cycle.

The ease of magnetic recovery of CalB@cap and the absence of enzyme leaching prompted us to investigate the catalytic recyclability of the MOF-based microcapsules. Following reaction of **1** and **2** for 8 h, the capsules were recovered, washed and used again for further transesterification reactions. Conversion yield of **3** reveals very good overall recyclability with 80% of activity maintained after six catalytic cycles (Fig. 4), comparing well to other Pickering-based microreactors with encapsulated CalB.^17^ In the present case, given the intact nature of the capsules and demonstrable heterogeneous nature of the catalysis, we attribute the modest decrease in conversion to the cumulative effects of enzyme denaturing by the 1-butanol substrate.^30^

For transesterification between larger substrates **4** and **5** (Fig. 3d), conversion to **6** with CalB@gel and CalB@Pick was similar to that observed for **3**, reaching 100% after ∼12 h (Fig. 3e and S27–S31†) In this case however, very little conversion was observed with CalB@cap for the first 12 h of reaction, although after this point a slow conversion to **6** was observed reaching 7.5% after 48 h. The observed induction period suggests a significantly increased diffusion barrier is provided by the capsule shell and/or potential blockage of pores or defects by larger molecules such as **4–6**. Specific activity for the controls followed the same trend as for **3**; however, with larger substrates the activity for CalB@cap is effectively zero during the early linear part of the conversion-time profile where specific activity is typically calculated (Fig. 3f). Given that **3** and **6** are formed by CalB@gel and CalB@Pick with very similar activity, the clear differences observed for CalB@cap are directly attributed to the variation in molecular size of both the substrates and products and the ability of the intact (microporous) capsule shell to regulate their access. The limiting molecular dimensions for the smaller substrates are 4.06 and 4.18 Å for **1** and **2**, respectively, and 4.06 Å for product **3** (Fig. S17†). These sizes are only slightly larger than the aperture size of ZIF-8 (3.4 Å), and given the uptake of 1-butanol (**1**) has previously been reported for this framework^31^ it seems likely that these molecules could pass through the micropores of the dense ZIF-8 outer layer to access the capsule interior. For the larger molecules **4–6**, the limiting molecular cross-section is 8.84 Å (Fig. S17†) so these are expected to be excluded by the micropores of the capsule shell. While two recent reports suggest that oversize molecules such as caffeine^32^ and Rhodamine 6G^33^ can be encapsulated by ZIF-8 arising from framework expansion or dissociative linker exchange mechanisms, it is most likely that **4–6** pass through very small pin-hole defects within the dense microporous ZIF-8 outer layer which is responsible for the observed albeit very low activity.

Although size-selective biocatalysis has previously been reported for oxidation reactions catalyzed by myoglobin immobilized within the mesoporous cages of a hierarchically porous MOF,^26^ the present work is a clear proof-of-principle demonstration that encapsulation of biomolecules using Pickering-stabilised hydrogels is a viable alternative strategy for the preparation of highly porous and functional MOF-based biocatalytic systems.

## Conclusions

In summary we have developed a synthetic route to prepare hierarchically porous MOF-based capsules around Pickering-stabilized hydrogels for the effective encapsulation of functional biomolecules, and demonstrated their practical utility in size-selective and recyclable biocatalysis. Processing of MOFs beyond simply increasing the framework pore size in the manner reported here, appears to be a very effective strategy to prepare highly porous functional MOF-biomolecule composite materials with significant potential in wider biotechnology applications including drug delivery, tandem chemoenzymatic catalysis and in novel bioseparation and biodegradation media.
