# Supplementary material for: Magnetic MOF microreactors for recyclable size-selective biocatalysis
Source: Chem Sci. 2014 Dec 17;6(3):1938–43. doi: 10.1039/c4sc03367a (PMC5501100; doi:10.1039/c4sc03367a)

Electronic Supplementary Material (ESI) for Chemical Science.
This journal is © The Royal Society of Chemistry 2014

Supporting Information

Magnetic MOF microreactors for recyclable size-selective biocatalysis

*Jia Huo, Jordi Aguilera-Sigalat, Samir El-Hankari and Darren Bradshaw**

S1 Materials and general methods

S2 Synthetic details

S3 Supplementary figures

S4 Calibration curves

Section S1: Materials and general methods.

Zinc nitrate ($Zn(NO_3)_2{\bullet}6H_2O$, 99%), zirconium tetrachloride ($ZrCl_4$, anhydrous, 99.99%), 2-methylimidazole (99%), 1,4-benzenedicarboxylic acid (H_2BDC, 98%) paraffin oil (puriss grade), Fluorescein isothiocyanate isomer I (FITC, >90%), rhodamine B (>95%), agarose (Type IX-A, ultra-low gelling temperature), Bradford reagent, Lipase B Candida Antarctica recombinant from *Aspergillus oryzae* (CalB, 9 U/mg), β-Galactosidase from *Escherichia coli* (β-Gal, > 500 U/mg), 1-butanol (>99.4%), vinyl acetate (>99%), 3-(4-hydroxyphenyl)propan-1-ol (>97%), and vinyl laurate (>99%) were all purchased from Sigma-Aldrich; Iron Oxide (II,III) (30 nm) was from Alfa Aesar; Green Fluorescent Protein (GFP) was from Enzo Life Sciences (UK) Ltd. All organic solvents are reagent grade and used without further purification, and water used in all experiments was dispensed from a Milli-Q Reagent Water System. A James SONIC4500 ultrasonic cleaner was used to disperse MOF nanoparticles in the oil phase. Pickering emulsions were formed using a shear force created by an IKA Ultra-Turrax T10 homogeniser. A Biopharma VirTis BT2KES freeze dryer was used to dry FITC-labelled enzymes.

Optical microscopy (OPM) measurements were carried out on a Leica M1750 M optical microscope. **Scanning electron microscopy** (SEM) measurements were made on a JEOL JSM 6500 thermal field emission scanning electron microscope at an accelerating voltage of 5, 10, or 15 kV. Samples for SEM measurements were prepared by firstly placing a drop of capsule suspension in absolute ethanol on a silica wafer attached to an aluminium substrate with a carbon paste that is dried under vacuum overnight, and then sputter-coated with a thin layer of conductive gold to improve electrical conductivity. For **confocal microscopy** studies, samples were incubated in a solvent containing a fluorescent dye (FITC in methanol or rhodamine B in H_2O) or the reaction solvent (2-butanol) and mounted on glass slides with a cavity, which was subsequently sealed with a cover slide. Images were acquired using a Leica SP2 confocal microscope (Leica, Mannheim, Germany) coupled to a Leica DMIRE2 inverted microscope (Wetzlar, Germany). The detection wavelengths were 486 and 543 nm for FITC and rhodamine B, respectively.

UV/vis spectra were collected with a SHIMADZU UV-2700 UV/vis spectrometer.

Powder X-ray diffraction patterns were collected on a Bruker D2 phaser in the angular range 2θ = 5-40° employing a Ni Kβ filter (detector side) producing Cu (Kα1/Kα2) radiation.

^{1}H-NMR spectra were acquired using a Bruker DPX400 FT-NMR spectrometer.

Fourier Transform Infrared (FTIR) spectra were collected on a Magna IR-560 Nicolet FTIR spectrometer equipped with a Mercure Cadmium Tellure detector. All experiments were run on a

horizontal attenuated total reflectance (ATR) crystal (ZnSe) where powders were pressed. Content of metals was analysed using a Varian Vista MPX

ICP-OES system by MEDAC Ltd. Samples for ICP were prepared by firstly destroying 5 mg microcapsules with 3 ml HCl (2 M) and 20 μl HF (48 % water solution) with sonication and then taking 2 ml of the supernatant for ICP after the above suspension was centrifuged at 10000 rpm for 10 min.

Thermogravimetric analysis (TGA) was performed using a TG 209 F1 Libra (Netzsch) and the sample was heated from room temperature to 900 ^{o}C at a rate of 10 ^{o}C min^{-1} under an air atmosphere.

N_2 adsorption/desorption isotherm was measured at 77 K using a Micromeritics 3-Flex Surface Characterization Analyzer after the sample was first degassed at 100 °C overnight. Surface areas were determined by the BET method in an appropriate pressure range, and total pore volume was determined using the adsorption branch of N_2 isotherm curve at the $p/p^0 = 0.99$ single point. Pore size distribution was determined using the adsorption branch of N_2 isotherms. Micropore size distribution analysis was carried out using the Horvath-Kawazoe method. Mesopore size distribution was calculated using the Barrett-Joyner-Halenda (BJH) method.

Section S2: Synthetic details.

Particle and Pickering emulsion preparation

UiO-66 nanoparticles were pre-hydrophobised with heptanoic acid through post-ligand exchange according to our previous method (Huo et al, *Adv Mate*r 2013, **25**, 2717) (named as Hep-UiO-66). Fe_3O_4 nanoparticles were also hydrophobised with heptanoic acid through post-treatment following minor modification of the method reported by Barbreta et al. (V. B. Barbeta, R. F. Jardim, P. K. Kiyohara, F. B. Effenberger, L. M. Rossi, J Appl Phys 2010, 107, 073913-073917) (named as Hep-Fe_3O_4).

A typical preparation procedure for UiO-66/Fe_3O_4 nanoparticle-stabilized agarose solution droplets (Pickering emulsions) is described as follows: Hep-UiO-66 (0.0288 g) and Hep-Fe_3O_4 (0.0032g) (9:1 wt/wt) nanoparticles were dispersed in 4 ml of liquid paraffin through sonication in an ultrasound bath for 10 min. 1.5 wt% agarose solution was prepared by dissolving 0.015 g of agarose in 1 g of PBS buffer (pH = 7.4) at 50 ^{o}C as stock solution. After cooling to 25 °C, agarose solution (200 μL) was added to the paraffin dispersion of UiO-66/Fe_3O_4 and homogenized under shear stirring at 11400 rpm for 2 min. The resulting Pickering phase was stored at 4 ^{o}C overnight to ensure full gelation of the nanoparticle-stabilized droplets.

Microcapsule preparation

A typical procedure to prepare ZIF-8 microcapsules is as follows: firstly, 2 x 250 ml stock solutions of $Zn(NO_3)_2$ (25 mM) were prepared in isopropanol and 2-butanol, and 2 x 250 ml stock solutions of 2-methylimidazole (2-MeIm) in isopropanol (100 mM) and 2-butanol (75 mM).

1ml of UiO-66/Fe_3O_4 nanoparticle-stabilized agarose solution droplets were immersed in a fresh mixture of 5 ml $Zn(NO_3)_2$ stock solution in isopropanol followed by addition of 5 ml 2-MeIm stock solution in isopropanol and left for 2 h at -20 ^oC before washing with fresh isopropanol. After the above procedure was repeated twice to stabilize the emulsion, the final ZIF-8 microcapsules were produced by immersing the obtained particles in a fresh mixture of 5 ml $Zn(NO_3)_2$ stock solution in 2-butanol followed by addition of 5 ml 2-MeIm stock solution in 2-butanol and left to stand for 1.5 h at -20 ^oC before washing with fresh 2-butanol. This step was repeated 8 times to ensure a dense intergrowth of ZIF-8 at the capsule exterior.

Rhodamine B loaded capsules

Rhodamine B-loaded microcapsules were prepared according to the above procedure using an agarose solution containing rhodamine B (0.5 mg/ml).

Rhodamine B loaded magnetic Pickering emulsions

Rhodamine B loaded magnetic Pickering emulsions were prepared according to the above procedure except only Fe_3O_4 nanoparticles were used to stabilize the droplets.

Release property studies

To evaluate the release properties, Rhodamine B-loaded ZIF-8 microcapsules (5 mg) were soaked in 3 ml of water, and a sample (1 ml) of supernatant was removed and centrifuged at time intervals of 1, 2, 3, 5, 8, 12, and 24 h. The concentration of rhodamine B released from the microcapsules was measured using a SHIMADZU UV-2700 UV/vis spectrometer by monitoring the absorbance of the aqueous solution at 557 nm. After each measurement was complete, the solution was returned to the sample vial to maintain the same overall volume. The total loading of rhodamine B within the capsules was calculated as 0.014 μmol/g by destroying ZIF-8 capsules with 3 ml of 0.2 M HCl aqueous solution containing 20 μl HF (48 % water solution). Under these conditions the MOF components are fully dissolved, leaving a slight white precipitate identified as the H_2BDC linker that was removed by filtration.

Uptake property studies

To evaluate the uptake properties, microcapsules were exposed to a bulk aqueous solution containing rhodamine B (10 mg/ml) or an ethanolic solution containing FITC (10 mg/ml) for 24 hrs. Confocal microscopy was used to monitor any uptake by the capsules.

Labelling of enzymes with FITC

35 μl of FITC solution in DMF (10 mg/ml) was added into 1 ml of an enzyme solution (1 mg/ml) in PBS buffer (pH = 7.2), and the solution shaken for 2 hrs at 200 rpm at ambient temperature. Free FITC was removed using a Sephadex G-25 column (pre-equilibrated and extracted with PBS buffer), and monitored by UV-vis spectroscopy. The samples were dried in a freeze dryer and then dissolved into 1 ml of PBS buffer for the subsequent preparation of FITC-labelled enzyme containing microcapsules.

Enzyme (or FITC labelled enzyme) loaded capsules

CalB (or β-Gal, or GFP)-loaded microcapsules were prepared according to the above procedure for the preparation of microcapsules, employing a 1.5 wt% agarose solution containing the biomolecule (10 mg/ml for CalB and β-Gal; 0.1 mg/ml for GFP). FITC-labelled enzyme-loaded capsules were prepared using the same method at a concentration of 0.5 mg/ml of CalB-FITC or β-Gal-FITC. While alcohols can be toxic to the enzyme resulting in partial denaturing, the ^{i}PrOH and 2-BuOH employed for ZIF-8 shell formation in the present case are reported to play less of a deactivating role (see Chen and Wu, *J Biosci Bioeng*. 2003, **95**, 466) hence the enzyme remains active following complete encapsulation under these conditions.

Enzyme loading calculation: Bradford method

5 mg of CalB-loaded microcapsules were ground with a mortar then destroyed with 3 ml of 0.2 M HCl aqueous solution and 20 μl HF (48 % water solution), followed by centrifugation at 12000 rpm for 10 min. 0.1 ml of the supernatant was mixed with 3 ml of Bradford reagent, which was incubated at room temperature for 15 min prior to UV measurement.

To minimize potential errors arising from the low pH, 0.1 ml of an identical acidic solution to that used for capsule degradation was mixed with 3 ml of Bradford reagent for standardisation. The difference of intensity at 595 nm of the sample and the standard was used to calculate the concentration of CalB within the MOF capsules.

Assessment of enzymatic activity of CalB-loaded microcapsules and control samples in organic solvents

The catalytic performance of CalB was determined via the transesterification between two series of substrates in acetone: small substrate, 1-butanol and vinyl acetate; and large substrates, 3-(4-hydroxyphenyl)propan-1-ol and vinyl laurate. As for small substrates, typically, a substrate solution (1 mL) containing 1-butanol (150 mmol/L) and vinyl acetate (100 mmol/L) in acetone was added into a vial containing CalB-loaded microcapsules (5 mg). The transesterification reactions were carried out on a shaker at 200 rpm under ambient conditions for a total of 48 hrs. After a defined interval, a 60 µL aliquot of the solution was removed and the concentration of the product analysed via ^{1}H-NMR. One unit of CalB activity (U) is defined as 1 µmol product produced per min and the specific activity is related to the normalized amount of the respective enzyme (U/mg). The activity of all samples was evaluated under the same conditions. The same procedure was followed for the larger substrates, except substrate concentration was reduced to 60 mmol/L to ensure complete solubility in the reaction solvent.

Section S3: Supplementary figures.

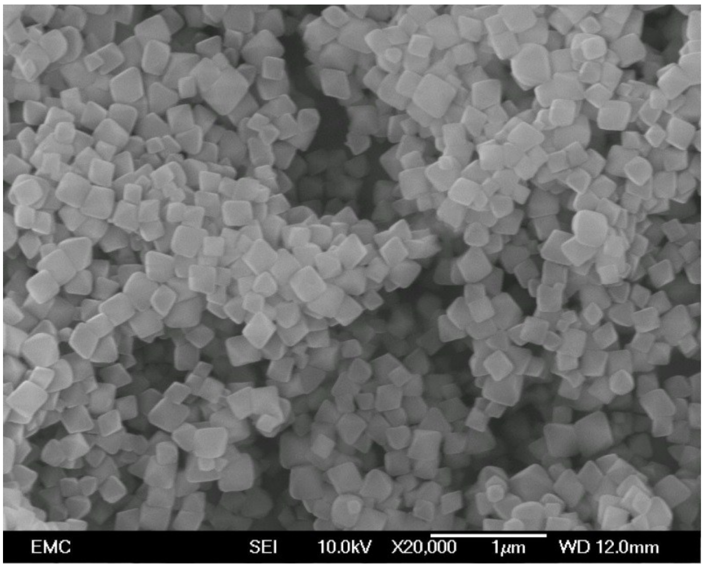

Figure S1. SEM image of the heptanoic acid functionalised UiO-66 nanoparticles used for Pickering stabilisation of the agarose hydrogel droplets.

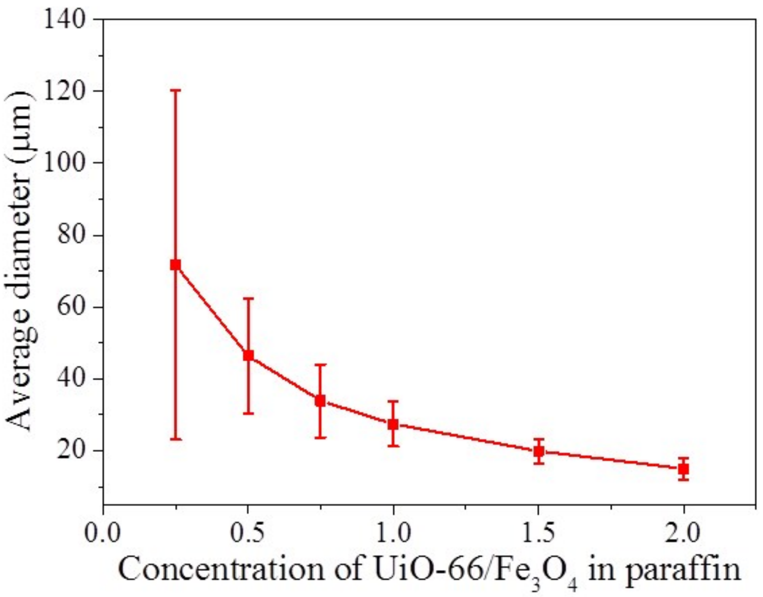

Figure S2. Plot of emulsion droplet size vs. amount of added UiO-66/Fe_3O_4 nanoparticles (at a constant 9:1 wt/wt ratio) by wt% with respect to the volume of the droplet phase.

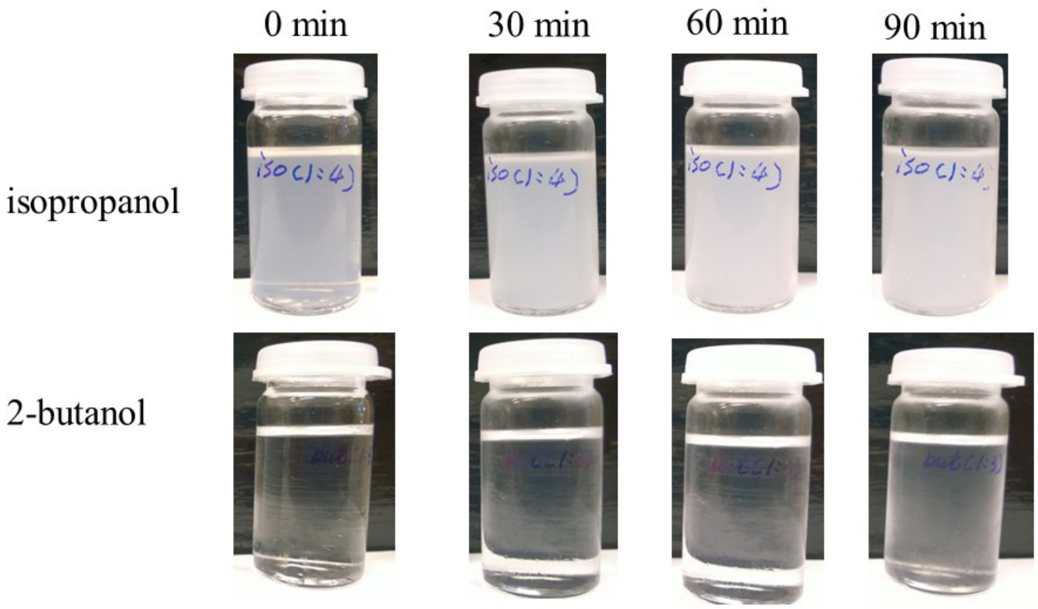

Figure S3. Extent of ZIF-8 formation with time following mixing of $Zn(NO_3)_2$ and 2-MeIm in isopropanol (^iPrOH) and 2-butanol (2-BuOH) solvents at -20°C as used for capsule shell formation. In ^iPrOH ZIF-8 immediately forms, but there is no discernible precipitation in 2-BuOH after 90 mins consistent with rapid nucleation and slow growth, respectively.

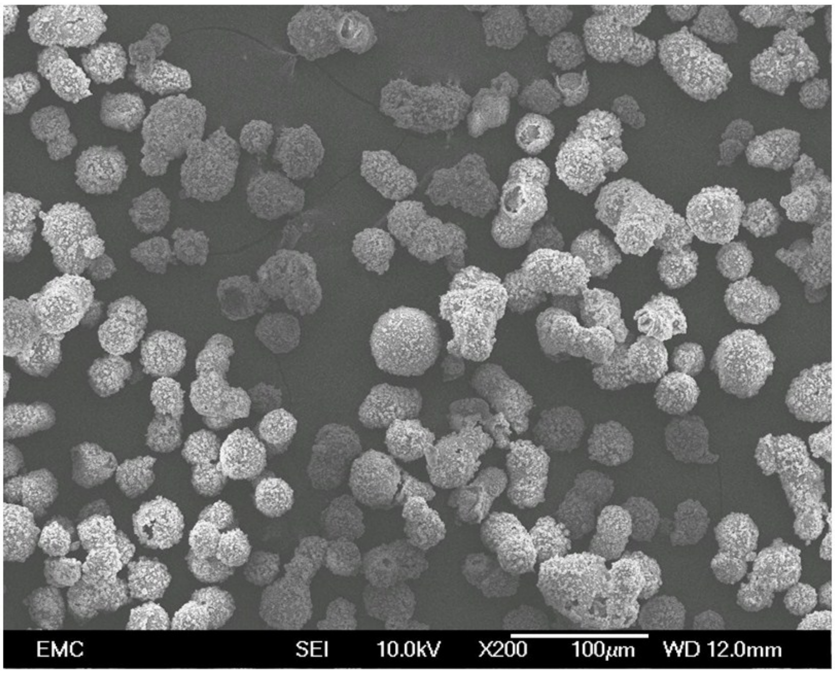

Figure S4. SEM image showing a number of fractured capsules following step one only of the synthesis, revealing their hollow nature. These capsules are more fragile than those following the complete two step shell formation and hence it is easier to observe broken/hollow structures.

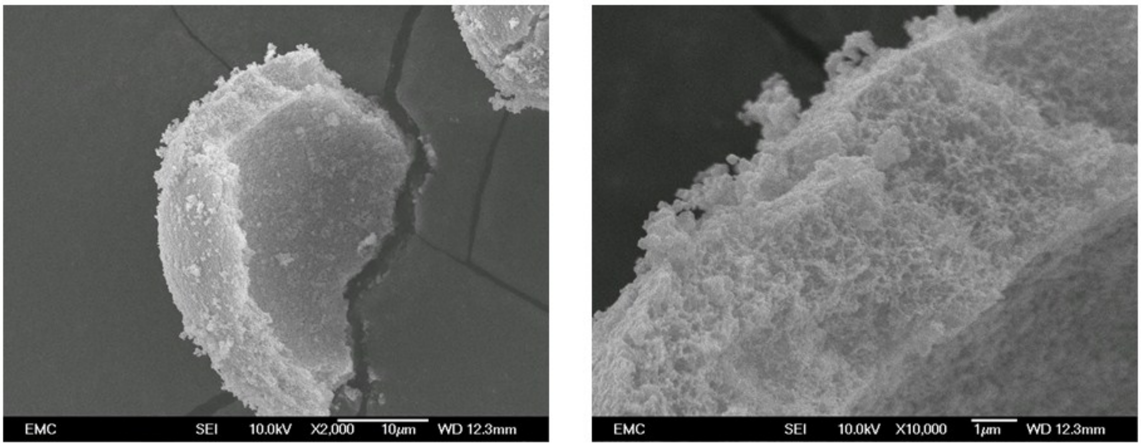

Figure S5. SEM images of the shell of a broken capsule following step one only of the synthesis which clearly reveal its network-like structure and absence of a dense surface layer.

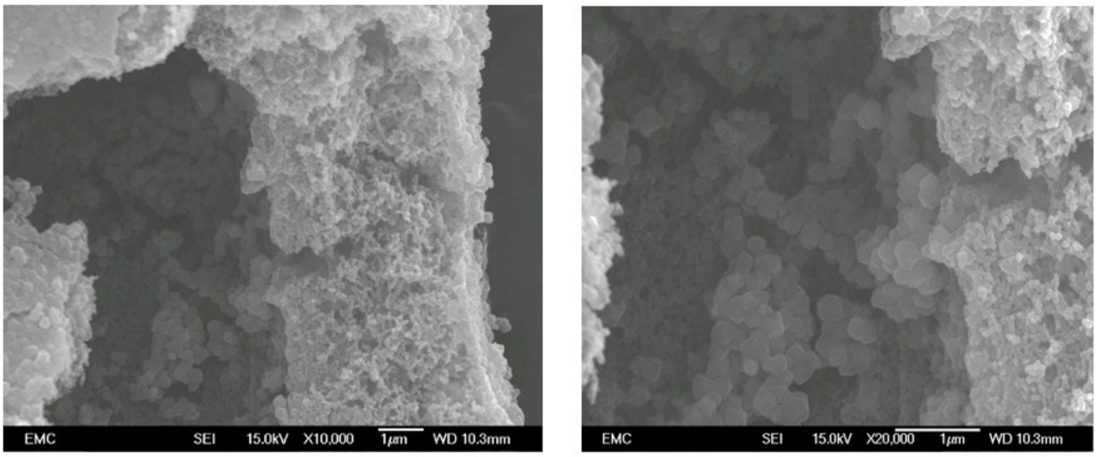

Figure S6. SEM images of the shell and interior of a broken capsule which further confirm the hierarchical nature of the ZIF-8 shell, and reveal the presence of UiO-66 NPs clustered around the interior of the shell wall. The UiO-66 NPs were used to stabilise the emulsion, and comparison to figure S1 shows these have retained their size and shape thus remaining unaffected during formation of the ZIF-8 shell.

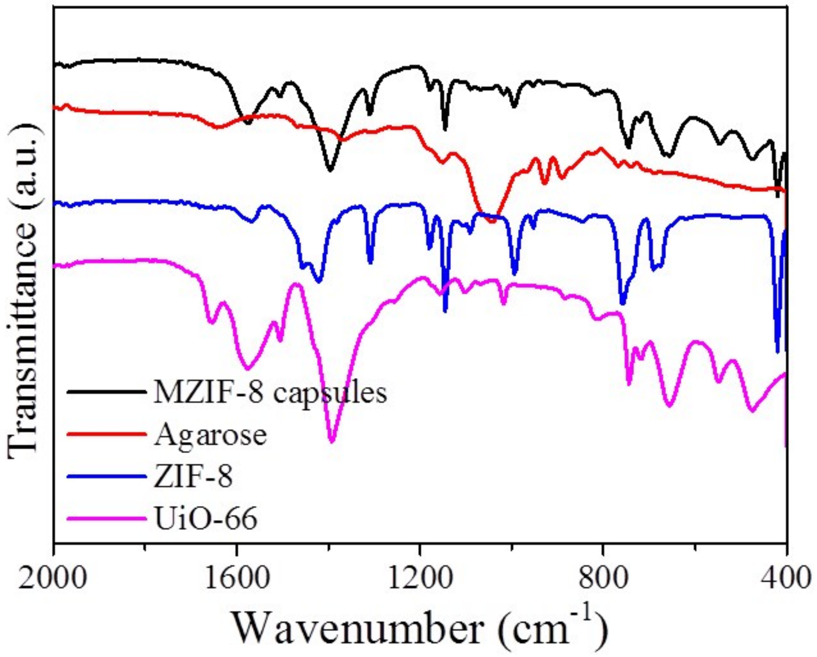

Figure S7. FTIR data of the capsules compared to spectra of the individual components indicating the presence of molecular vibrations for both the ZIF-8 shell and the UiO-66 particles used to stabilize the agarose hydrogel cores.

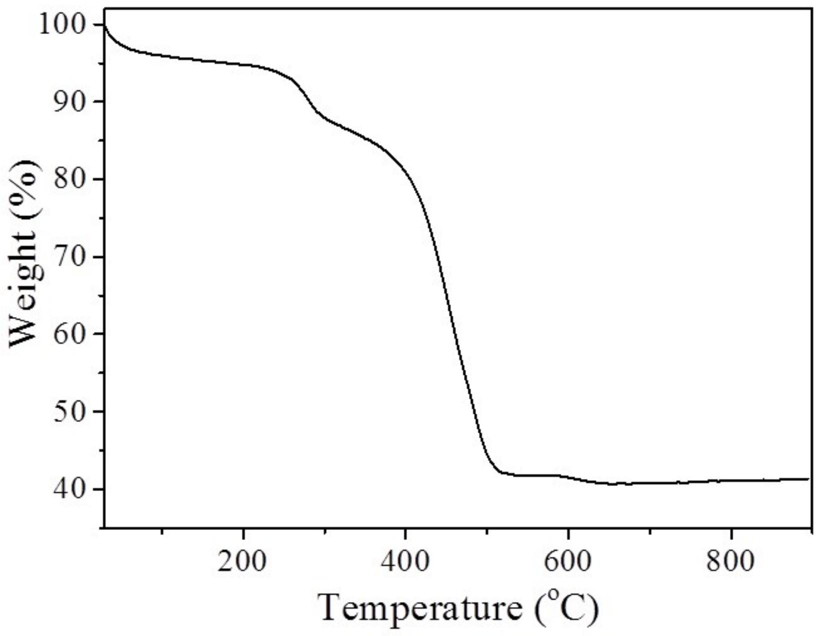

Figure S8. TGA profile of the MOF microcapsules. The most significant mass loss is observed between 320-500°C, commensurate with capsule decomposition within this range. A small step is also observed between 500-650°C which we cannot attribute with certainty due to the complex composition of the capsules which contain heptanoic-acid functionalised UiO-66, ZIF-8, agarose and Fe_3O_4 (also surface-functionalised with heptanoic acid).

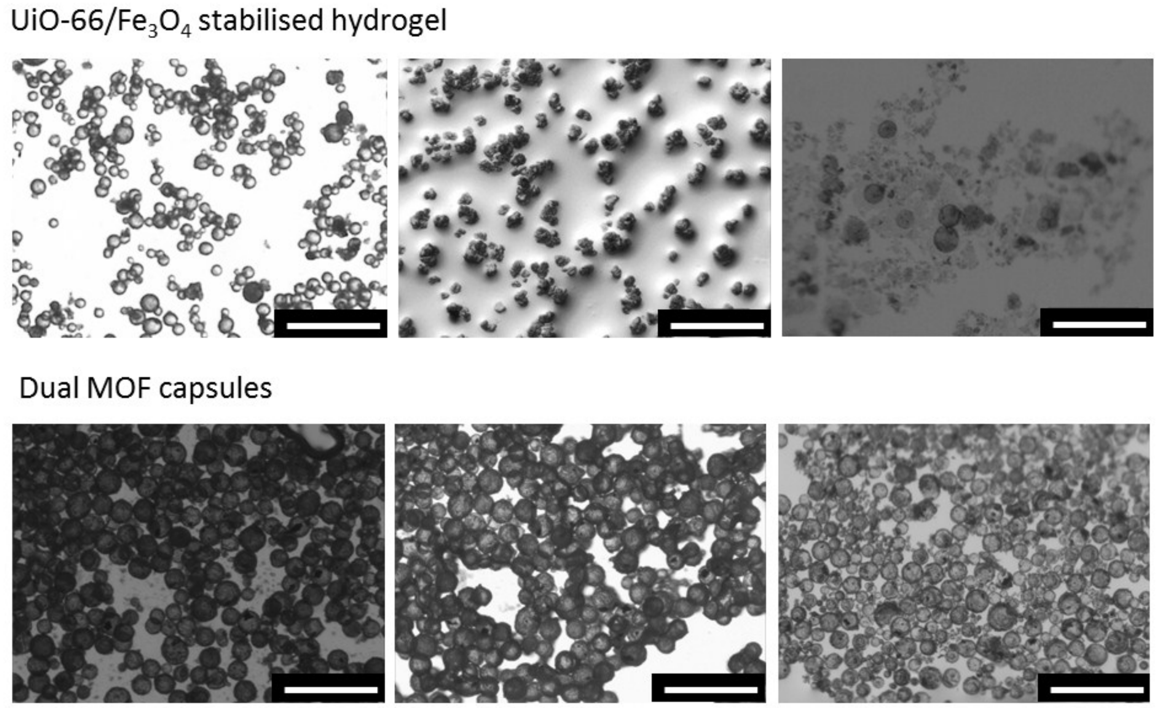

Figure S9. Optical micrographs of the Pickering-stabilised hydrogel and the MOF capsules in hexane (left), after solvent removal (middle) and following rehydration (right) demonstrating the highly robust nature of the microcapsules to solvent exchange and removal. Scale bar = 200 µm

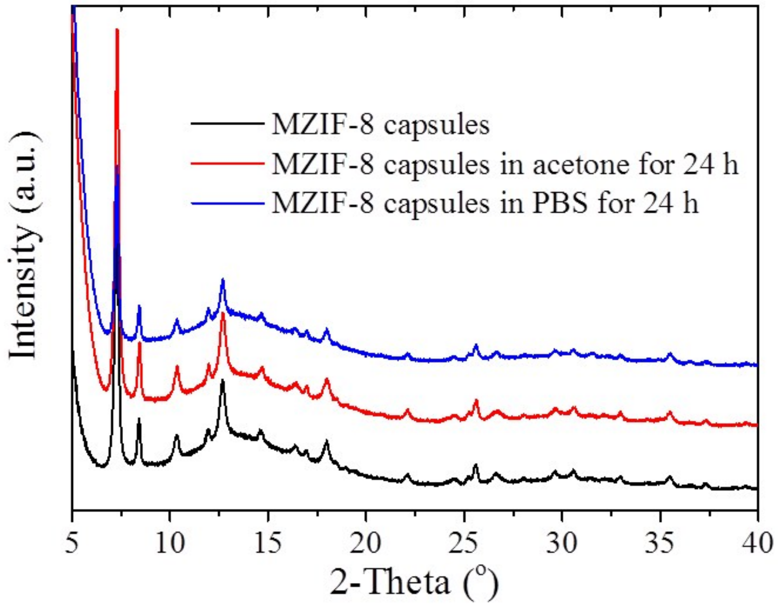

Figure S10. PXRD data of the microcapsules after soaking in various media for 24 hrs revealing the stability of the MOF shell. These data are entirely consistent with the shell composition of the capsules: both ZIF-8 and UiO-66 are highly stable framework configurations.

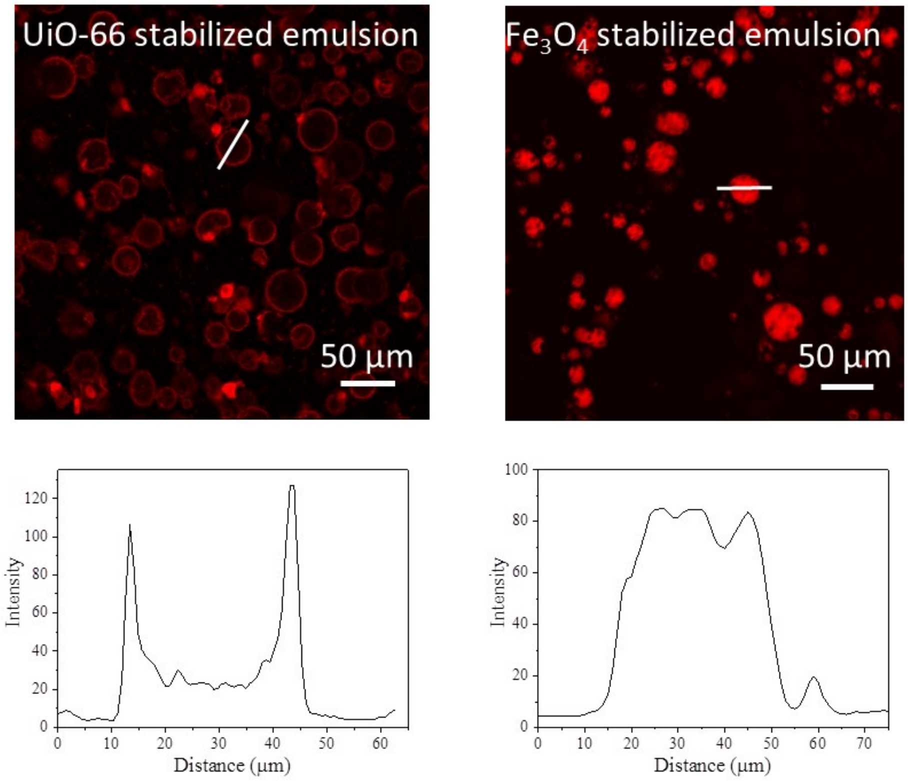

Figure S11. CLSM images of RhB loaded hydrogels Pickering-stabilized by particles of UiO-66 or Fe_3O_4. The intensity profiles shown below correspond to the cross-section of the highlighted capsules. These images reveal the role of the MOF in driving the accumulation of the dye around the interior surface of the capsules.

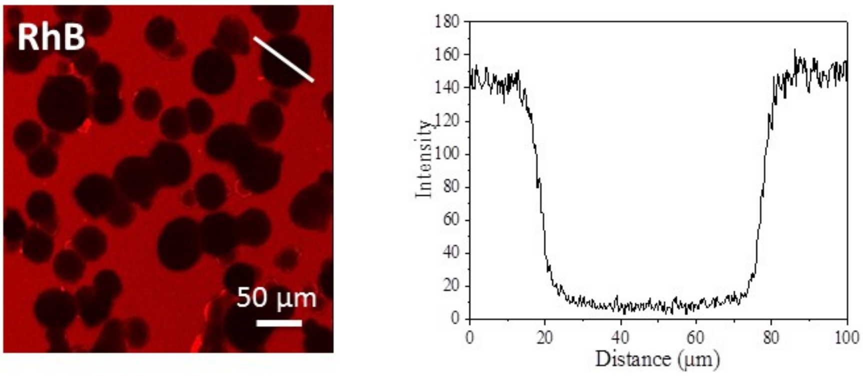

Figure S12. CLSM image and typical fluorescence intensity profiles of capsules soaked in an aqueous solution of RhB, further demonstrating the exclusion of the dye by the dense microporous layer at the capsule exterior.

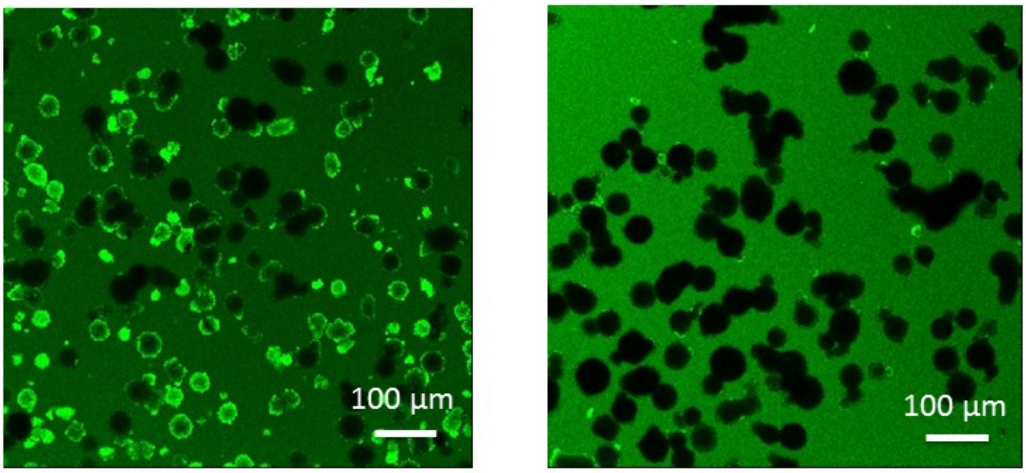

Figure S13. CLSM images of microcapsules following soaking in methanolic FITC solutions after stage one of the synthesis only (left) where a network-like shell is present (see figure S3), compared to those with the dense overlayer (right). Uptake of FITC through the porous network is clear and confirms the role of the microporous exterior in controlling access through the capsule shell.

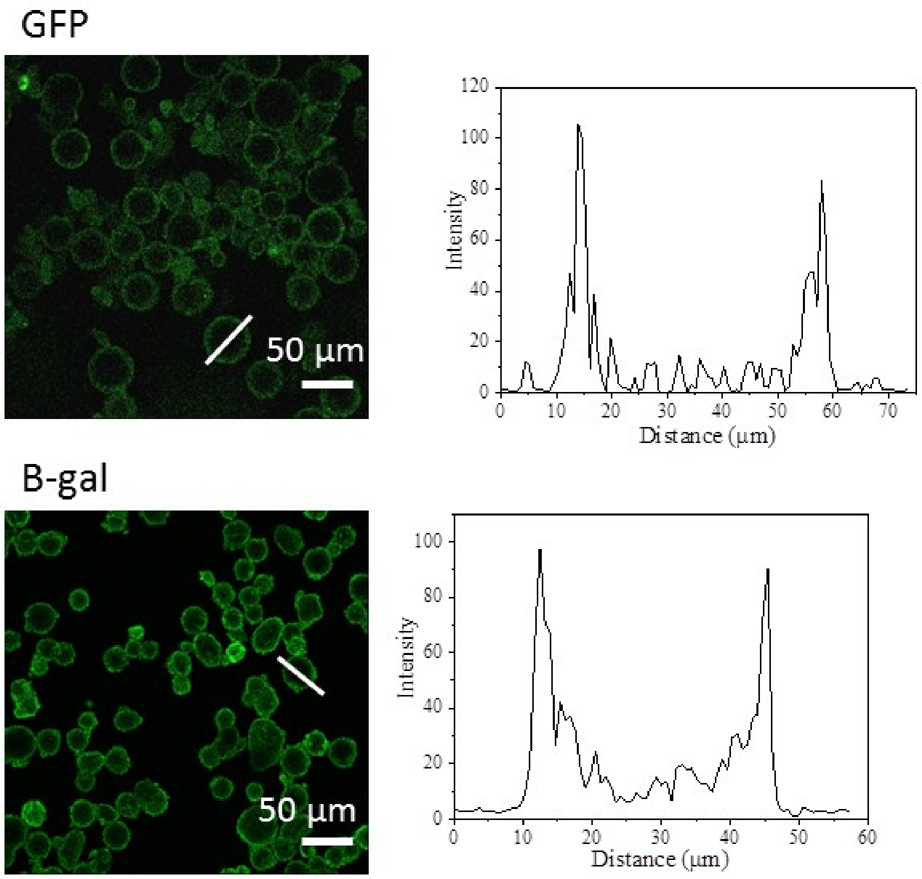

Figure S14. CLSM images and intensity profiles for encapsulated biomolecules: green fluorescent protein (top) and FITC labelled β-galactosidase (bottom).

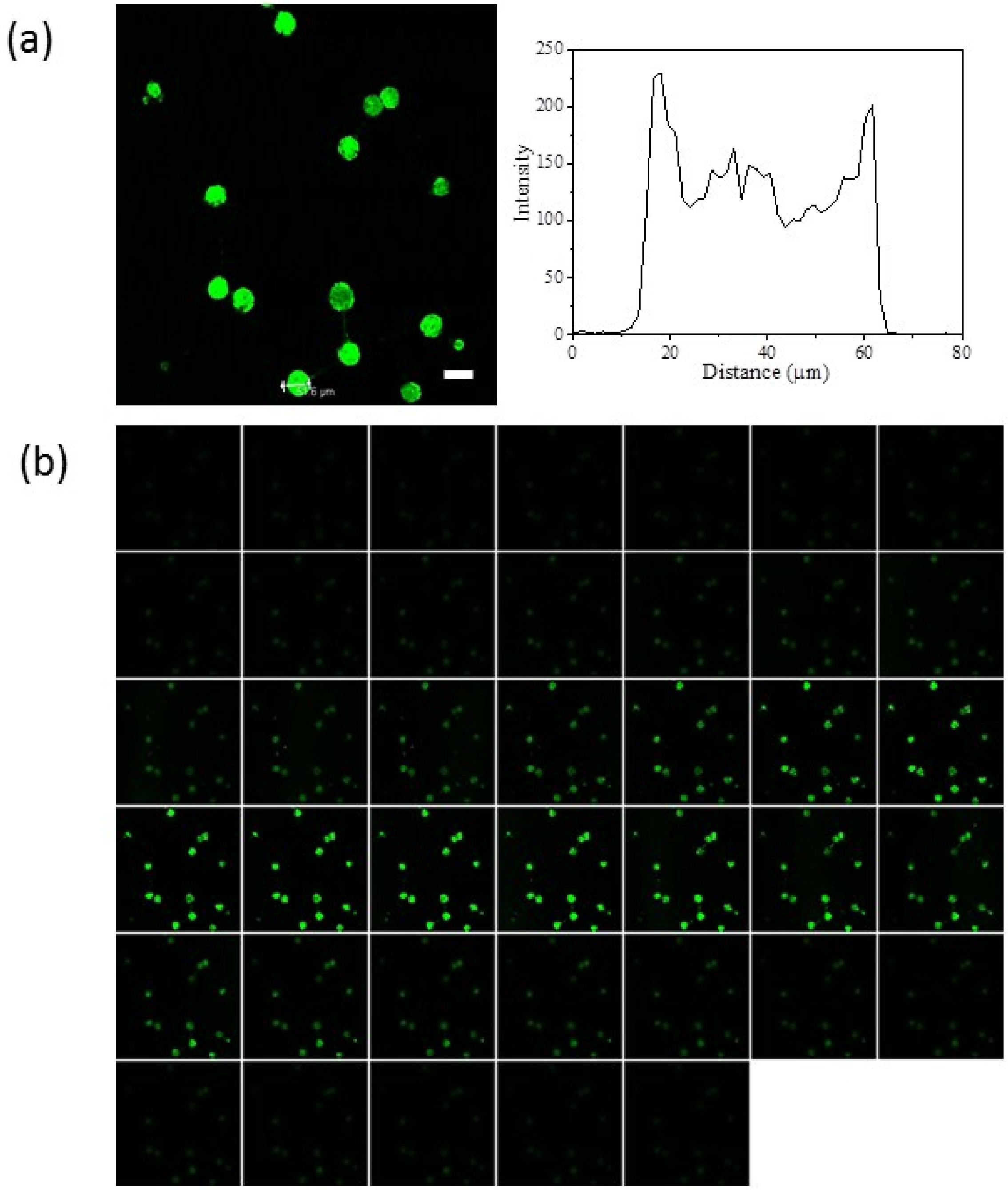

Figure S15. (a) CLSM image and intensity profile of FITC-CalB loaded capsules following soaking in methanol overnight. (b) Individual slices taken through the capsules at height intervals of 2.6 μm showing the presence of the enzyme homogeneously distributed in the capsule lumen. The greatest fluorescence intensity is of course recorded for those slices passing closest to the centre of the capsules.

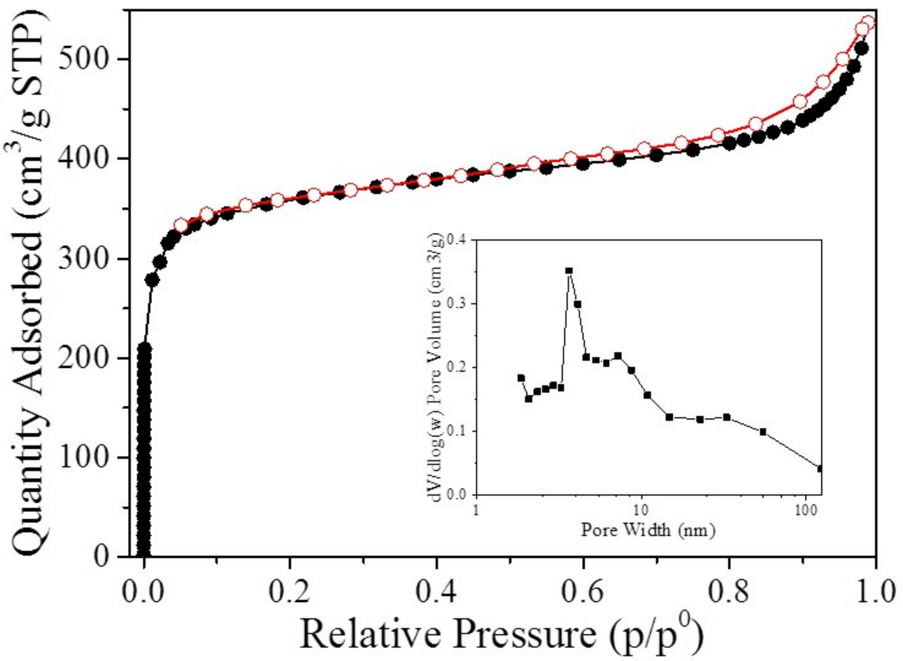

Figure S16. Nitrogen adsorption-desorption isotherm of CalB loaded microcapsules (CalB@cap) at 77 K (black squares, adsorption; red squares, desorption). The inset shows the BJH pore size distribution calculated from the adsorption branch of the isotherm.

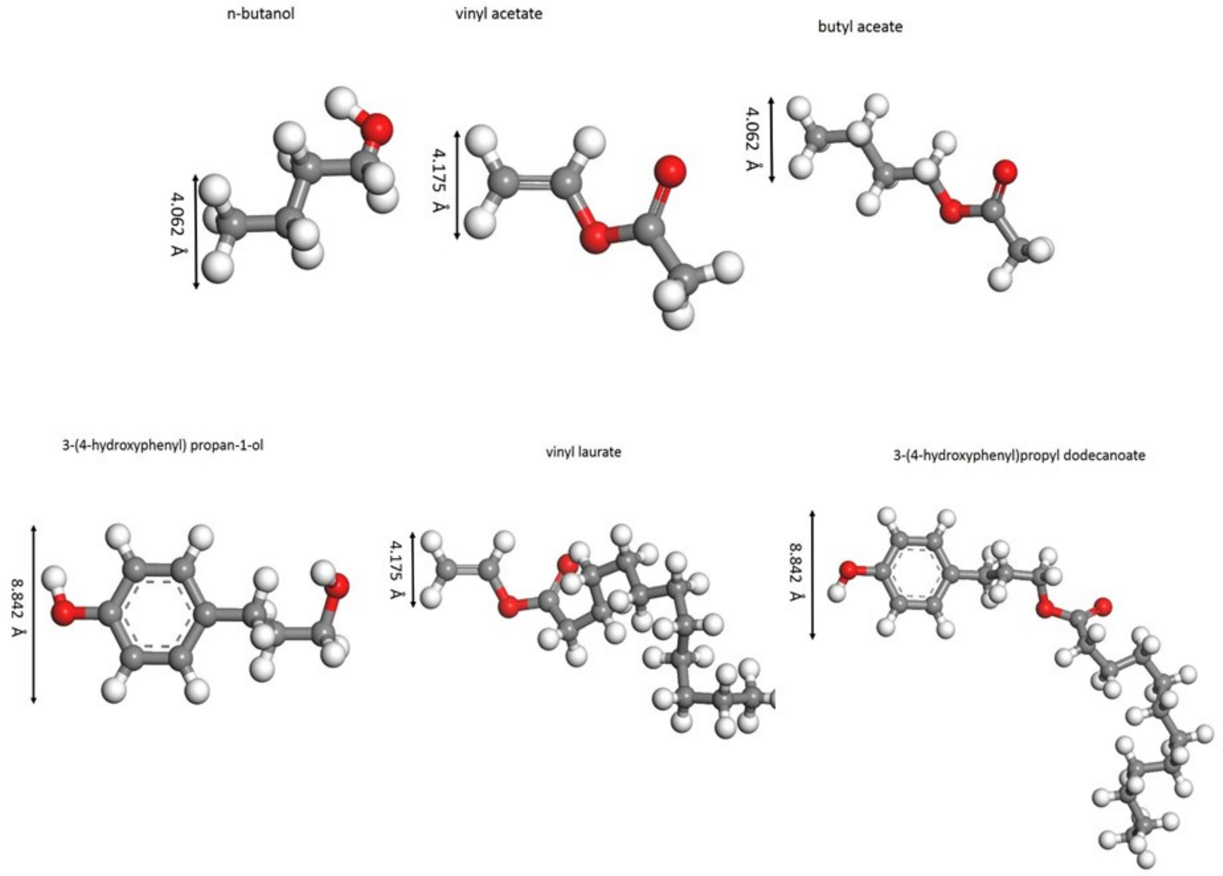

Figure S17. Molecular cross sections of the substrates used and products formed following biocatalysis with CalB@ZIF-8 microcapsules.

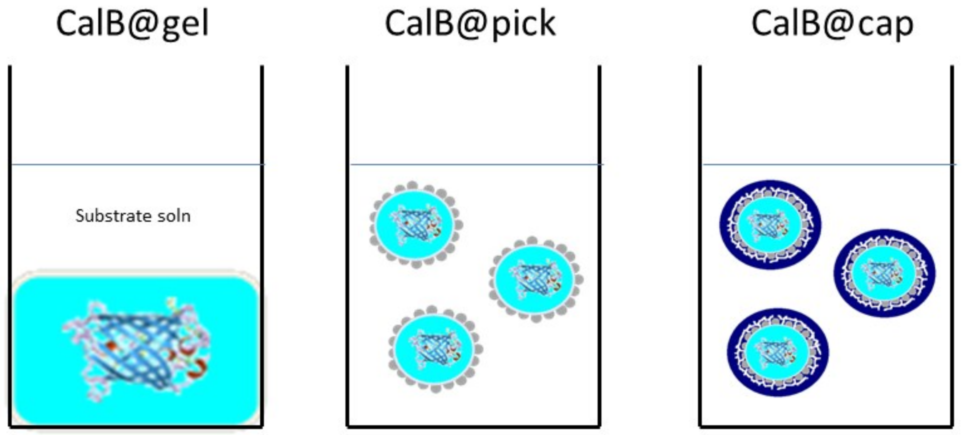

Figure S18. Schematic illustration of the controls used for biocatalysis by the enzyme-loaded capsules. CalB@gel is an agarose monolith with the substrate solution layered above; CalB@pick is composed of hydrogel droplets Pickering stabilised by UiO-66/Fe_3O_4 nanoparticles; CalB@cap are the capsules with the hierarchically structured MOF shell described in the text. There is an increasing surface area for reaction moving L-R in the figure. Note: the enzymes shown are for representation only and do not reflect the relative amounts of biomolecules incorporated into the real systems.

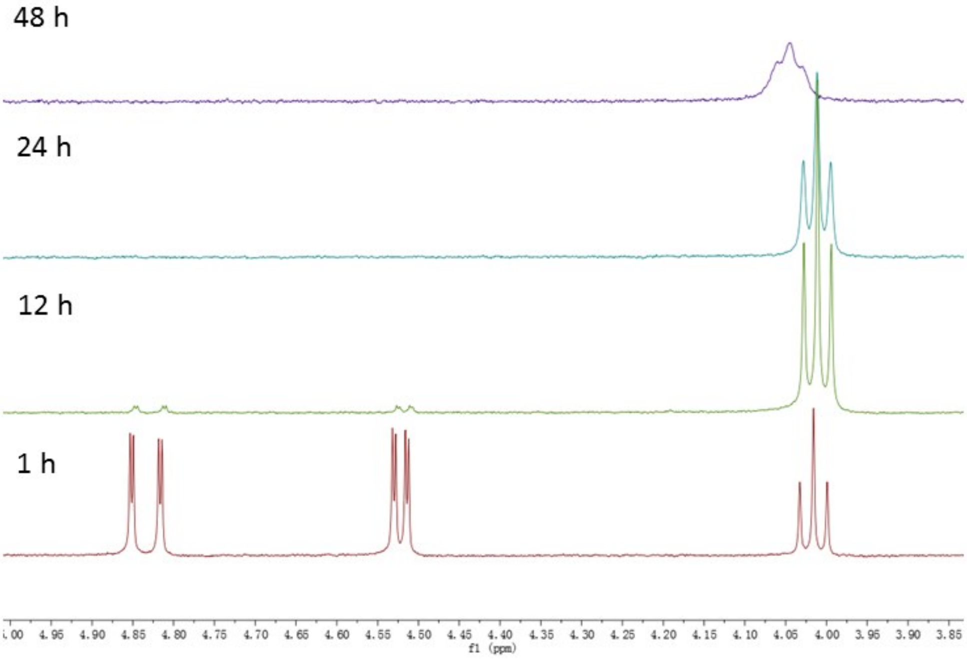

Figure S19. ^{1}H NMR spectra for the transesterification reaction between **1** and **2** yielding product **3** (butyl acetate) catalysed by CalB@pick. Spectra are shown at representative time intervals, and conversion is based on the ratio between vinyl-containing substrates and products: depletion of the vinyl group of substrate **2** at 4.5 and 4.8 ppm vs. the appearance of the triplet corresponding to the methylene group adjacent to the carbonyl ($CH_3OCO\mathbf{CH_2}CH_2CH_2CH_2$) at 4.02 ppm in product **3**.

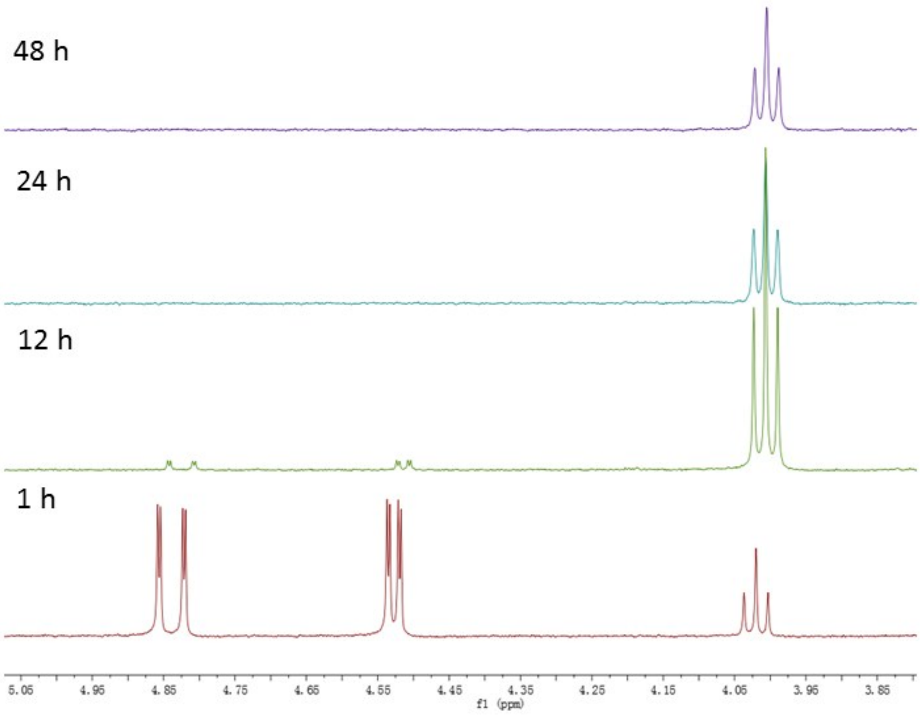

Figure S20. ^{1}H NMR spectra for the transesterification reaction between **1** and **2** yielding product **3** (butyl acetate) catalysed by CalB@gel.

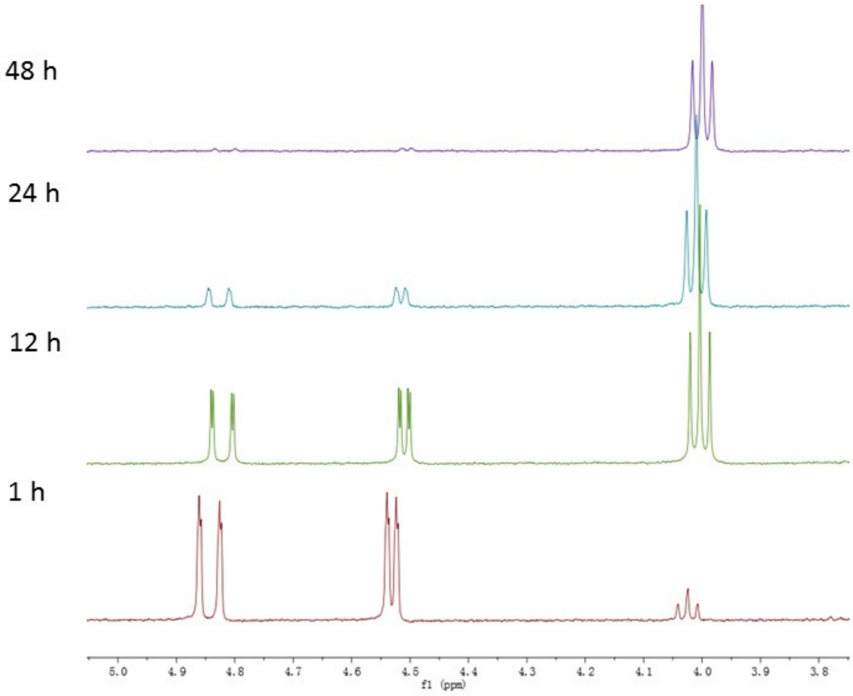

Figure S21. ^{1}H NMR spectra for the transesterification reaction between **1** and **2** yielding product **3** (butyl acetate) catalysed by CalB@cap.

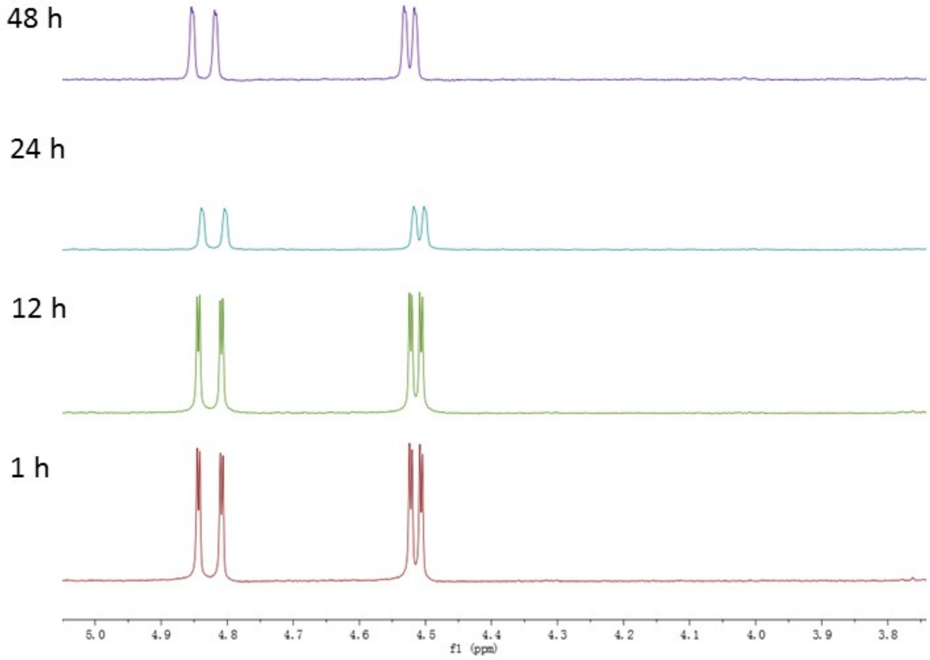

Figure S22. ^{1}H NMR spectra for the transesterification reaction between **1** and **2** yielding product **3** (butyl acetate) catalysed by capsules only (cap) in the absence of CalB.

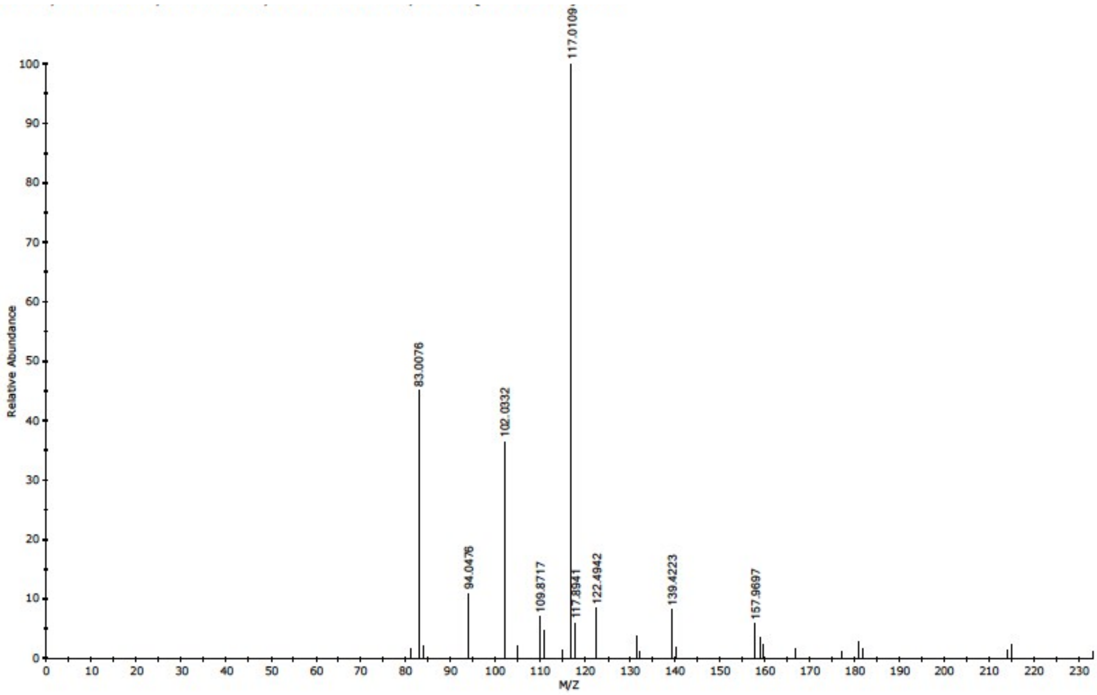

Figure S23. ESI-MS of the reaction product following the transesterification reaction between **1** and **2** catalysed by CalB@cap confirming the presence of butyl acetate.

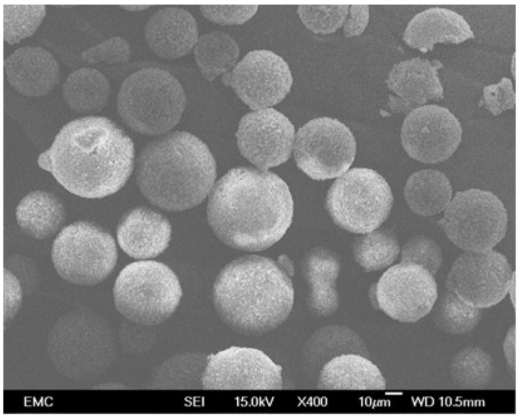

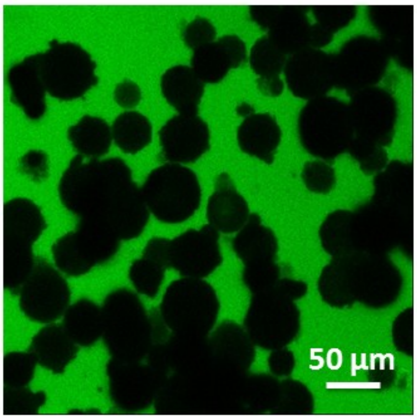

Figure S24. (left) SEM image of capsules following the transesterification reaction between **1** and **2**; (right) CLSM image of the same capsules following soaking for 24 hrs in a MeOH solution of FITC.

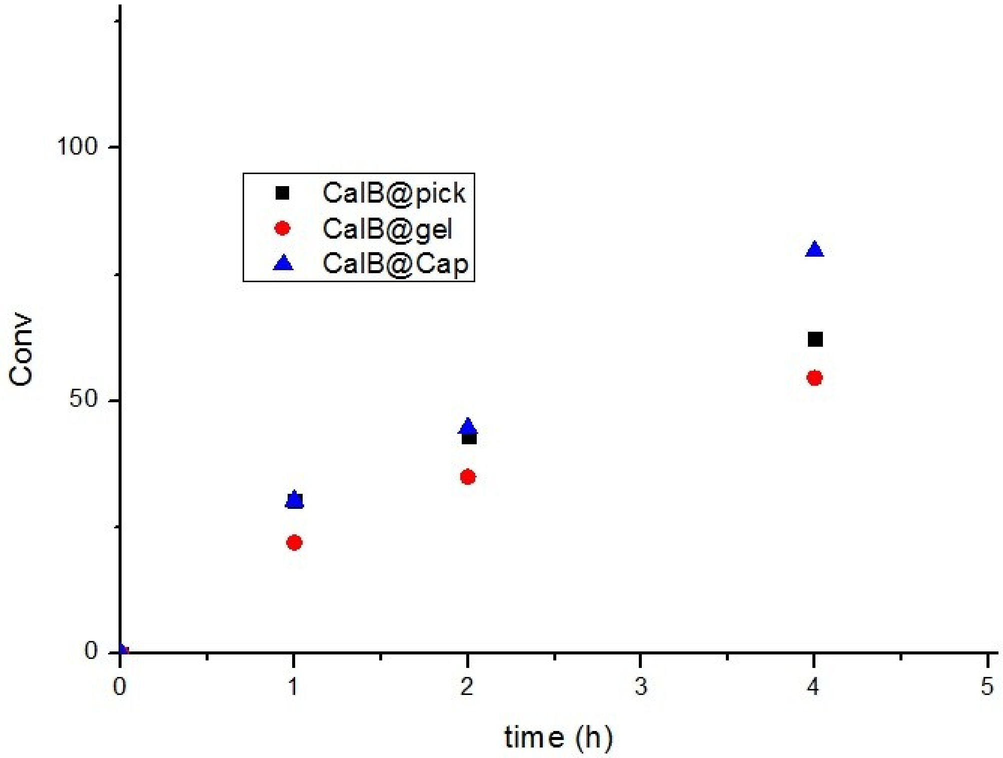

Figure S25. % conversion to product **3** vs. time, normalized to the total amount of protein determined in each system by Bradford assay. The controls are shown schematically in figure S14.

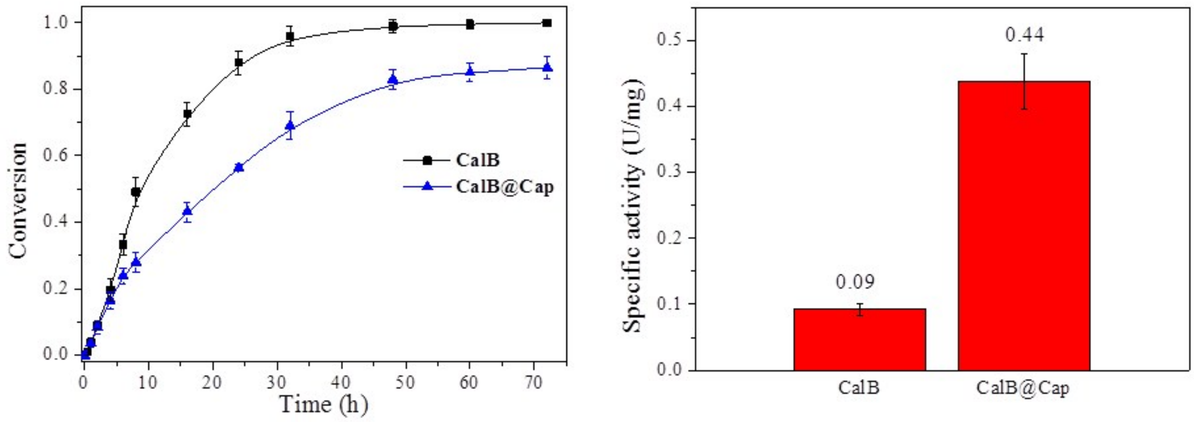

Figure S26. (left) conversion-time profile and (right) specific activities of free CalB and CalB@cap for the transesterification of **1** and **2** in hexane.

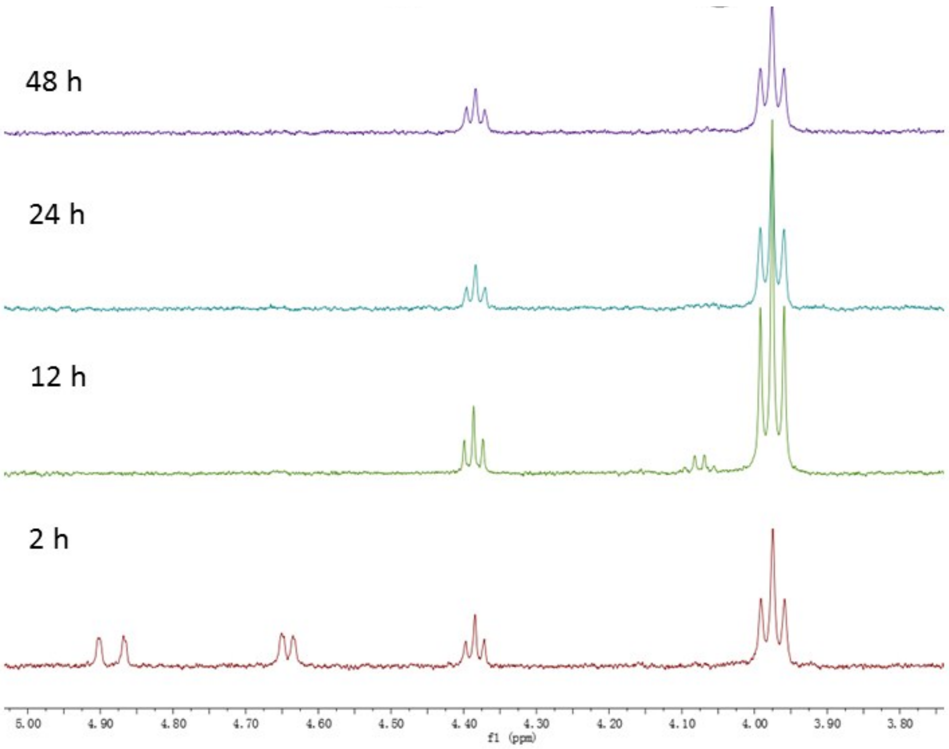

Figure S27. ^{1}H NMR spectra for the transesterification reaction between **4** and **5** yielding product **6** (3-(4-hydroxyphenyl)propyl dodecanoate) catalysed by CalB@pick. Spectra are shown at representative time intervals, and conversion is based on the ratio between vinyl-containing substrates and products: depletion of the vinyl group of substrate **5** at 4.90 and 4.65 ppm vs. the appearance of the triplet corresponding to the methylene group adjacent to the carbonyl at 3.97 ppm in product **6**.

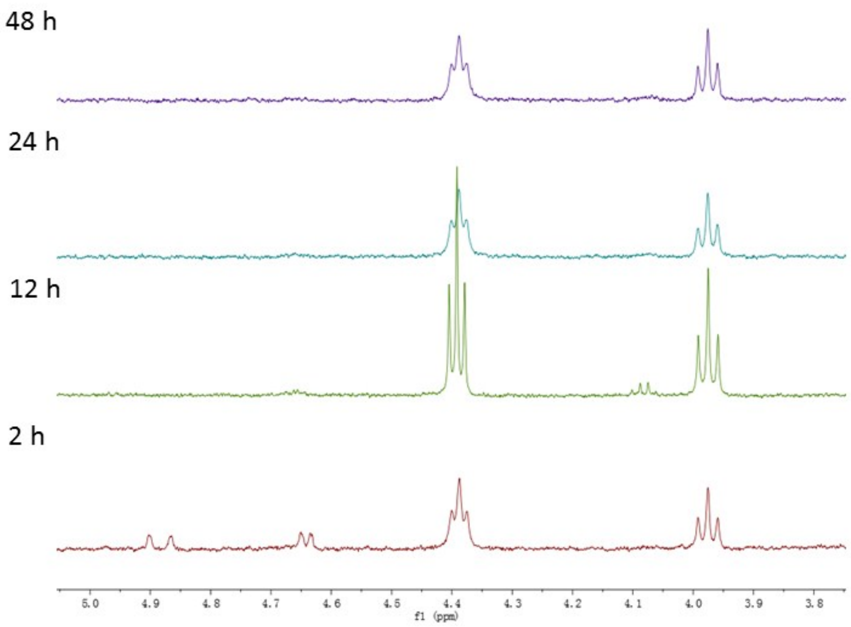

Figure S28. ^{1}H NMR spectra for the transesterification reaction between **4** and **5** yielding product **6** (3-(4-hydroxyphenyl)propyl dodecanoate) catalysed by CalB@gel.

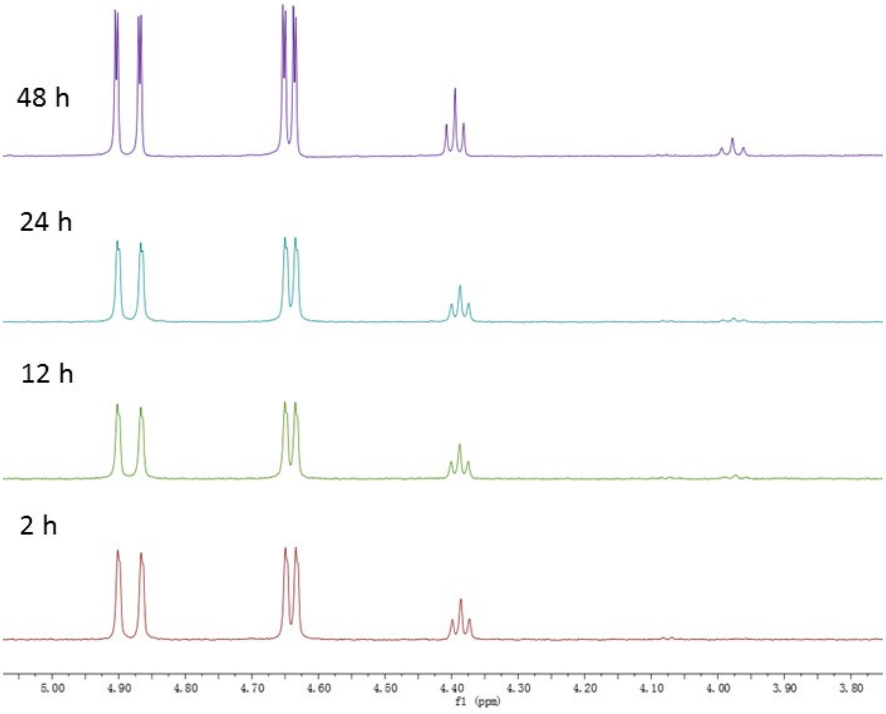

Figure S29. ^{1}H NMR spectra for the transesterification reaction between **4** and **5** yielding product **6** (3-(4-hydroxyphenyl)propyl dodecanoate) catalysed by CalB@cap.

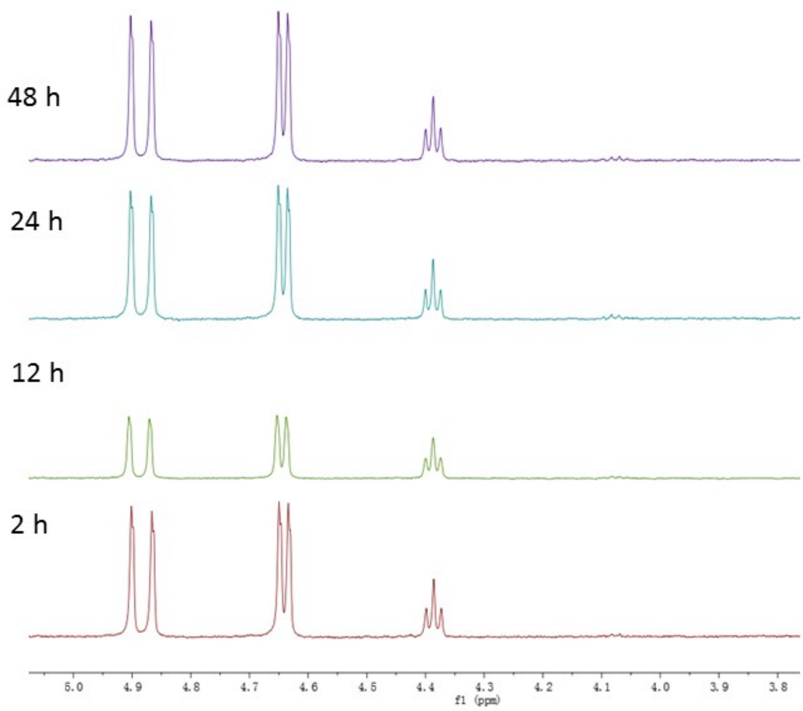

Figure S30. ^{1}H NMR spectra for the transesterification reaction between **4** and **5** yielding product **6** (3-(4-hydroxyphenyl)propyl dodecanoate) catalysed by capsules only (cap) in the absence of CalB.

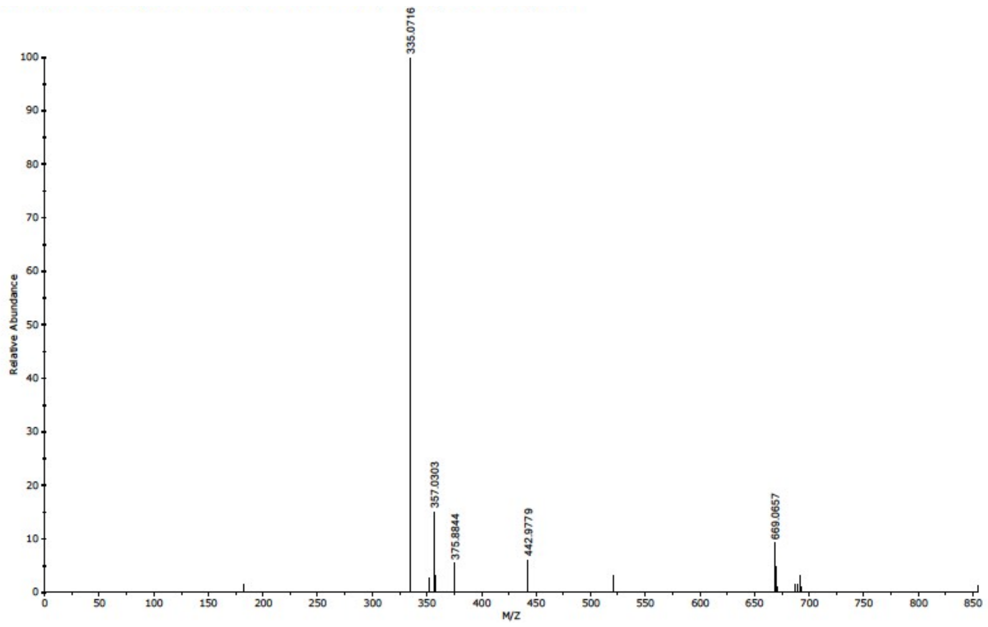

Figure S31. ESI-MS of the reaction product following the transesterification reaction between **4** and **5** catalysed by CalB@cap confirming the presence of 3-(4-hydroxyphenyl)propyl dodecanoate.

Section S4: Calibration curves.

1. Standard curve for RhB in aqueous solution

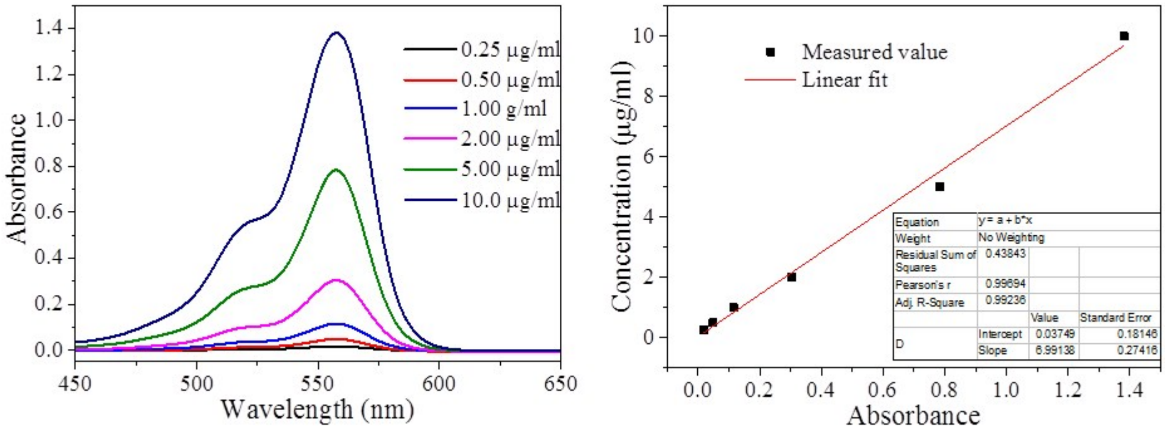

2. Standard curve for FITC in aqueous solution

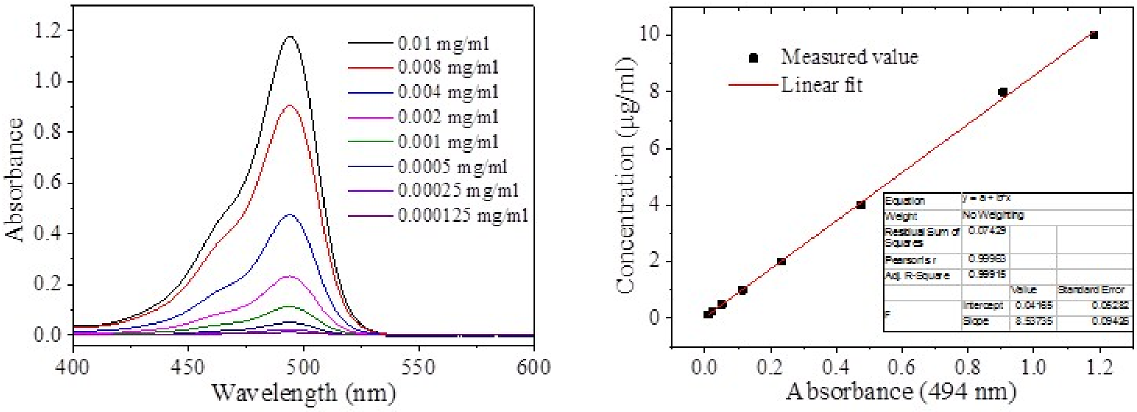

3. CalB loading standard curve by Bradford assay

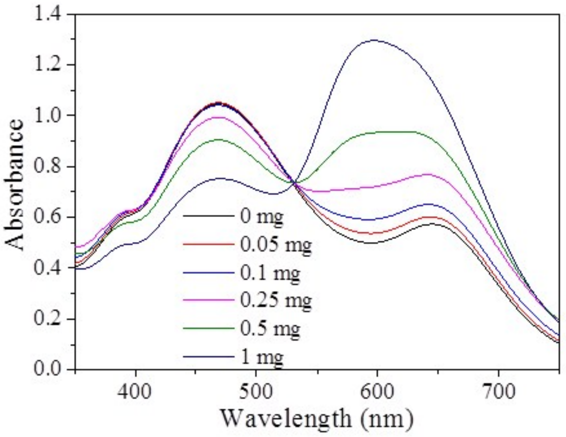

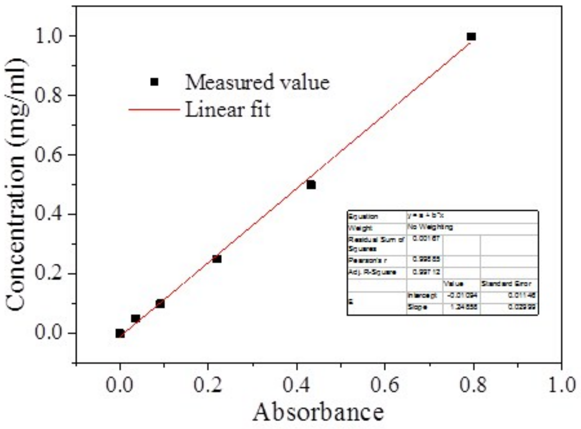

Supplement: Supplementary file 1 [file SC-006-C4SC03367A-s001.pdf]
